# *Phytophthora megakarya* and *P. palmivora*, Causal Agents of Black Pod Rot, Induce Similar Plant Defense Responses Late during Infection of Susceptible Cacao Pods

**DOI:** 10.3389/fpls.2017.00169

**Published:** 2017-02-14

**Authors:** Shahin S. Ali, Jonathan Shao, David J. Lary, Mary D. Strem, Lyndel W. Meinhardt, Bryan A. Bailey

**Affiliations:** ^1^Sustainable Perennial Crops Laboratory, United States Department of Agriculture/Agricultural Research Service, Beltsville Agricultural Research Center-West, Plant Sciences InstituteBeltsville, MD, USA; ^2^Physics Department, University of Texas at DallasRichardson, TX, USA

**Keywords:** cacao, black pod rot, *Phytophthora*, RNA-Seq, self-organizing map, plant-pathogen interaction

## Abstract

*Phytophthora megakarya* (Pmeg) and *Phytophthora palmivora* (Ppal) cause black pod rot of *Theobroma cacao* L. (cacao). Of these two clade 4 species, Pmeg is more virulent and is displacing Ppal in many cacao production areas in Africa. Symptoms and species specific sporangia production were compared when the two species were co-inoculated onto pod pieces in staggered 24 h time intervals. Pmeg sporangia were predominantly recovered from pod pieces with unwounded surfaces even when inoculated 24 h after Ppal. On wounded surfaces, sporangia of Ppal were predominantly recovered if the two species were simultaneously applied or Ppal was applied first but not if Pmeg was applied first. Pmeg demonstrated an advantage over Ppal when infecting un-wounded surfaces while Ppal had the advantage when infecting wounded surfaces. RNA-Seq was carried out on RNA isolated from control and Pmeg and Ppal infected pod pieces 3 days post inoculation to assess their abilities to alter/suppress cacao defense. Expression of 4,482 and 5,264 cacao genes was altered after Pmeg and Ppal infection, respectively, with most genes responding to both species. Neural network self-organizing map analyses separated the cacao RNA-Seq gene expression profiles into 24 classes, 6 of which were largely induced in response to infection. Using KEGG analysis, subsets of genes composing interrelated pathways leading to phenylpropanoid biosynthesis, ethylene and jasmonic acid biosynthesis and action, plant defense signal transduction, and endocytosis showed induction in response to infection. A large subset of genes encoding putative Pr-proteins also showed differential expression in response to infection. A subset of 36 cacao genes was used to validate the RNA-Seq expression data and compare infection induced gene expression patterns in leaves and wounded and unwounded pod husks. Expression patterns between RNA-Seq and RT-qPCR were generally reproducible. The level and timing of altered gene expression was influenced by the tissues studied and by wounding. Although, in these susceptible interactions gene expression patterns were similar, some genes did show differential expression in a *Phytophthora* species dependent manner. The biggest difference was the more intense changes in expression in Ppal inoculated wounded pod pieces further demonstrating its rapid progression when penetrating through wounds.

## Introduction

*Theobroma cacao* L. (cacao), the source of cocoa and a critical ingredient in chocolate, is grown around the world where favorable tropical environments occur. Black pod rot (BPR), caused by various species of *Phytophthora*, is the most important disease of cacao on a global scale with losses estimated at 700,000 metric tons in 2012 (Ploetz, 2016). *Phytophthora palmivora* (E. J. Butler) is present in most of the cacao growing countries around the globe and has a broad host range (McHau and Coffey, 1994). *Phytophthora megakarya* Brasier and Griffin (Pmeg) occurs only in the countries of West and Central Africa and is considered a significant pathogen only on cacao. Pmeg is the most virulent species in the *Phytophthora* genus causing BPR and can cause 60–100% crop losses if not managed (Opoku et al., 2000) whereas Ppal generally causes losses of 20–30% annually (Flood et al., 2004). Pmeg was first identified taxonomically as a species in 1979 (Brasier and Griffin, 1979). By the mid 1980's, Pmeg became predominant on cacao in Nigeria, Cameroon, Equatorial Guinea, Gabon, and Togo (Guest, 2007), was confirmed in Ghana in 1985 (Dakwa, 1987), and continues to spread (Ali et al., 2016). Ppal is no longer routinely isolated from cacao in Cameroon and Nigeria (Nyasse et al., 1999; Ndubuaku and Asogwa, 2006; Djocgoue et al., 2007) but how Pmeg has displaced Ppal from cacao in these countries is unclear. Ppal tends to have a more rapid growth rate than Pmeg in culture, possibly contributing to its ability to cause accelerated necrosis in mechanically wounded cacao tissues compared to Pmeg (Ali et al., 2016). In a susceptible cacao genotype, mechanical wounding is almost irrelevant for Pmeg infection (Ali et al., 2016). Pmeg and Ppal have estimated genome sizes of 126.88 and 151.23 Mb, respectively with 42,036 and 44,327 genes, respectively (Ali et al., in press). Ppal has gone through whole genome duplication and subsequent gene diversification has consequently expanded its genetic capacity for nutrient acquisition and breakdown of complex structures, for example cell walls. This capacity may influence Ppal's vigorous growth and broad host range, even without extended co-evolution with cacao. Pmeg on the other hand, has undergone amplification of specific gene families, some of which are clearly virulence-related like RxLRs, CRNs, elicitins and NPPs (Ali et al., in press). During *Phytophthora* infection, appressoria release effectors even before penetrations that enter host cells in an attempt to suppress PAMP (pathogen-associated molecular pattern)-triggered immunity (Giraldo and Valent, 2013).

Quite a few studies have focused on the plant-specific reactions of the *Phytopthora*/cacao interaction. In general, most of the studies focus on anatomical/histological differences, enzymes, genes, and metabolites that are influenced by interactions between *Phytophthora* and cacao genotypes varying in resistance to BPR (Bailey et al., 2015). Most of these studies have concentrated on aspects of polyphenol and lignin formation. Aspects of plant cellular structure are also thought to contribute to resistance/tolerance to BPR (Ndoumou et al., 1995). In addition, Nyadanu et al. (2012) found increasing thickness of epicuticular wax associated with tolerance to BPR in cacao. A better understanding of the genes involved in the biosynthesis and regulation of these various traits will be critical to optimizing breeding efforts to develop cacao genotypes providing sustainable tolerance to BPR.

Tolerance to *Phytophthora* is considered a quantitative trait with evidence to support at least 13 consensus QTLs and 8 genomic regions being involved (Lanaud et al., 2009). EST libraries from *Phytophthora* infected tissues were included in an exhaustive examination of the cacao transcriptome (Argout et al., 2008). The general consensus is that resistance to one *Phytophthora* species contributes resistance to all *Phytophthora* species (Bailey et al., 2015). Eventually, it should be possible to verify if all QTLs that contribute to resistance/tolerance to individual *Phytophthora* species also contribute resistance/tolerance to all *Phytophthora* species attacking cacao.

The observation that cacao genotypes show similar relative resistance/tolerance rankings regardless of the *Phytophthora* species being studied clearly suggests similar host factors contribute to resistance/tolerance regardless of the *Phytophthora* species involved. Yet, levels of resistance/tolerance are reduced against Pmeg compared to Ppal regardless of cacao genotype although relative genotype rankings of resistance/tolerance remain the same. Our objective was to use transcriptomic tools to begin dissecting the plant-specific reactions between Pmeg/Ppal and cacao starting with virulent isolates of both *Phytophthora* species and a susceptible cacao genotype. Through this research, we established a starting point for future studies of the diverse reactions of cacao genotypes showing resistance/tolerance to infection by *Phytophthora* species and contribute to understanding why Pmeg is the more virulent of the two species.

## Materials and methods

### *Phytophthora* isolates and inoculum preparation

*Pmeg isolates* Gh-ER1334 and Ca-ZTHO145, and Ppal isolates Gh-ER1349 and IC-SBR112.9 used in this study were isolated from BPR infected cacao in Ghana, Cameroon, and Côte d'Ivoire as described previously (Ali et al., 2016) and maintained on CV8 agar plate at 18°C. Zoospore inoculum was produced for various infection studies essentially as described by Lawrence (1978) and modified by Ali et al. (2016).

### Plant materials

To identify the cacao responses to Pmeg and Ppal infection, various plant inoculation studies were carried out. For all the pod husk and leaf disc inoculation assays, Catongo cacao trees susceptible to *Phytophthora*; Crouzillat et al., 2000) were maintained in a USDA-ARS, Beltsville, MD greenhouse at 60% relative humidity and a photoperiod of 12 h light at 25 ± 3°C and 12 h dark at 21 ± 3°C. Ambient light was supplemented with 400 W high-pressure sodium lamps, as needed, to obtain 250 μmol.m^−2^s^−1^. Automatic retractable shade cloths were used to limit light to a maximum of 1000 μmol.m^−2^s^−1^. Plants were drip-irrigated two times daily and fertilized regularly with a controlled-release complete fertilizer (Nutricote Total 18-6-8 Type 80, Arysta Life Science North America Corp., NC) at a rate of 670 g.m^−2^ every 12 weeks to maintain adequate levels of soil moisture and nutrition.

### Zoospore inoculated unwounded pod husk assay

Unwounded pod husk inoculation assays were carried out to assess the virulence of Ppal (isolate IC-SBR112.9 and Gh-ER1349) and Pmeg (isolate Ca-ZTHO145 and Gh-ER1334) essentially as described by Ali et al. (2016). Briefly, 4–5 month old pods were cut into 2.5 × 2.5 cm pieces; the inner core materials removed and dipped in sterile 0.7 mM benzimidazole (senescence retardant) solution to prevent browning until used. Husk pieces were surface-sterilized with 6% (vv^−1^) regular bleach (Clorox, USA) for 90 s followed by three rinses with sterile distilled water. Pieces were placed in sterile plastic containers (20 × 10 × 6 cm) lined with sterile tissue paper soaked in 0.7 mM benzimidazole solution at the bottom. For inoculation, 40 μl zoospore solutions were placed in the middle of the exterior part of each husk piece. Control husk pieces were treated with 40 μl sterile water. Containers were covered and incubated at 25°C with 50% RH and under 12 h light (200 lx) and dark cycles. Samples were photographed at 24 h intervals. The area under necrosis was quantified using Image J software (Abràmoff et al., 2004) and expressed as cm^2^. Observations were taken up to 4 days and the area under disease progress curve (AUDPC) was calculated (Shaner and Finney, 1977).

(1) $$\text{AUDPC}=\overset{n}{\underset{i\;=\;1}{\sum^{\text{​}}}}\left[{\left(Y_{i\;+\;1}+Y_{i}\right)}^{-2}\right]\left[t_{i\;+\;1}-t_{i})\right]$$

Where, *Y*_*i*_ is an assessment of a disease progression (cm^2^) at the *i*th observation, *t*_*i*_ is time (day) at the *i*th observation, and *n* is the total number of observations. Each experiment was repeated independently twice with three replicating husk pieces per isolate per experiment.

### Zoospore inoculated wounded pod husk assay

For wounded pod husk inoculation assays, husk pieces were treated and placed in sterile plastic containers as mentioned above. Husk surface was punctured with a no. 1 cork borer (5 mm diameter) and 2–3 mm deep holes were created which were subsequently filled with 40 μl zoospore solutions of Ppal (isolate IC-SBR112.9 and Gh-ER1349) and Pmeg (isolate Ca-ZTHO145 and Gh-ER1334). Controls were filled with 40 μl sterile water. Containers were covered and incubated at 25°C with 50% RH and under 12 h light (200 lx) and dark cycles. Samples were photographed at 1 day intervals and the progression of necrotic area was quantified using Image J software (Abràmoff et al., 2004) and expressed as cm^2^. Observations were taken up to 3 days and the AUDPC was calculated as mentioned above. Each experiment was repeated independently twice with three replicating husk pieces per isolate per experiment.

### Estimation of secondary inoculums load

The secondary inoculums loads were estimated based on the surface sporangia count of the infected pod husk pieces. At 5 days post inoculation (dpi), each infected pod husk piece was washed with 1 ml Tween-20 solution (2% v/v). After serial dilution, sporangia solutions were plated on 20% V8-PARP agar (Ferguson and Jeffers, 1999). From each replicating husk pieces, 24 single colonies were picked and transferred to a 24-well plate containing 2 ml liquid CV8 and grown at room temperature for 4–5 days. Mycelia from each well were picked with sterile tooth picks and transferred to 2 ml safe lock tube (Eppendorf, USA), followed by flash freezing in liquid nitrogen and freeze drying. DNA from each sample was extracted as mentioned before by Ali et al. (2016) and subjected to a Pmeg and Ppal specific PCR assay as described by Ali et al. (2016) to determine the % of secondary inoculums.

### Zoospore inoculated leaf disc assay

Stage 3 cacao leaves (light green) (Bailey et al., 2005) were harvested from Catongo cacao trees. Leaf discs with 1.5 cm diameter were cut out with No. 24 cork borer and placed on 90 mm Petri plate lined with Whatman no. 2 filter paper soaked with 0.7 mM benzimidazole solution. For inoculation, a 20 μl zoospore suspension of Ppal (isolate Gh-ER1349) and Pmeg (Gh-ER1334) was placed at the center of the leaf disc. Controls were treated with water alone. Petri plates were covered and incubated at 25°C with 50% RH and under 12 h light (200 lx) and dark cycles.

### RNA extraction from infected plant material

Infected pod husk and leaf discs were harvested at 1, 2, and 3 dpi and flash frozen in liquid nitrogen. For RNA isolation, samples were ground finely in mortar and pestle and transferred to a 50 mL centrifuge tube containing 15 mL of 65°C extraction buffer (Bailey et al., 2005). Additional extraction methods were conducted as previously described (Bailey et al., 2013).

### Transcriptome sequencing

RNA was extracted from unwounded pod husk inoculated with Ppal isolate Gh-ER1349 and Pmeg isolate Gh-ER1334. RNA-Seq analysis was carried out by the National Center for Genome Resources (Santa Fe, NM, USA). cDNA was generated using the RNA library preparation TruSeq protocol developed by Illumina Technologies (San Diego, CA). Using the kit, mRNA was first isolated from total RNA by performing a polyA selection step, followed by construction of single end sequencing libraries with an insert size of 160 bp. Single-end sequencing was performed using the Illumina HiSeq2000 platform. Samples were multiplexed with unique six-mer barcodes generating filtered (for Illumina adapters/primers and PhiX contamination) 1 × 50 bp reads. The sequences acquired by RNA-Seq were verified by comparison to the Pmeg and Ppal genomes assembled in this study. RNA reads from RNA-Seq libraries ranging from 50 to 70 million reads in fastq format were aligned using memory-efficient short-read aligner Bowtie-2–2.1.0 (Langmead and Salzberg, 2012) to the coding sequences (CDS) of the cacao genome (Argout et al., 2010). Tabulated raw counts of reads to each CDS, respectively, were obtained from the bowtie alignment. Estimation of normalized RPKM (Reads Per Kilobase of transcript per Million mapped reads) and statistical analysis of expression level using count data of each gene with three replicates for each library was performed using the DEseq package (Anders, 2010) and R x64 2.15.2 program (http://www.r-project.org/). Genes were grouped into four categories, either up or down regulated (≥2-fold) for each species. KEGG (Kyoto Encyclopedia of Genes and Genomes) Automatic Annotation Server (KAAS) was used to obtain KEGG Orthology and KEGG pathways involving the differentially expressed genes by BLAST comparisons against the manually curated KEGG GENES database (Moriya et al., 2007). Gene ontology (GO) analysis was carried out using the program Blast2GO (Conesa et al., 2005).

### Expression analysis using RT-qPCR

Procedures for RT-qPCR and analysis were as described by Bailey et al. (2006). Pathogen load in each sample was calculated by comparing expression of Pmeg/Ppal reference genes (*Pm*TP/*Pp*TP) to expression of three cacao reference genes (TcRefs) (*TcACT, TcGAPDH*, and *TcACP*) (See Supporting Excel File S1, Sheet A). The geometric mean (C_T•*Tc*Ref_) (Vandesompele et al., 2002; Ali et al., 2014) was calculated for cycle times of TcRefs (C_T•*Tc*Ref(1−3)_] and the ΔC_T_ was calculated for *Pm*TP/*Pp*TP (C_T•__*Pm*__TP/*Pp*TP_–C_T•*Tc*Refs_).

A total of 36 cacao genes were chosen for analysis by RT-qPCR. Nineteen cacao genes monitoring plant response during Pmeg/Ppal infection (See Supporting Excel File S1, Sheet A) were chosen based on their putative function and to represent a range of differential expression values between Pmeg, Ppal, and control treatments as observed in the RNA-Seq analysis to verify the RNA-Seq analysis. Another set of 17 genes was selected based on their putative participation in the plant hormone signal transduction pathway and plant-pathogen interaction pathway based on KEGG analysis. All 36 genes were analyzed across the three replicating sets of RNA from the Ppal isolate Gh-ER1349 and Pmeg isolate Gh-ER1334 infected and untreated control samples and analyzed for temporal and tissue specific transcriptions in response to Pmeg/Ppal infection in leaves and wounded and unwounded surfaces of pod husk pieces. The treatments include unwounded pod husks at 1 and 3 dpi, wounded pod husks at 1 dpi, and leaf disc at 1 and 2 dpi. Each treatment had three replicated samples for untreated controls, Pmeg, and Ppal infection. To obtain the relative transcript levels, the threshold cycle (C_T_) values for all genes of interest (C_T•GOI_) were normalized to the geometric mean (Vandesompele et al., 2002; Ali et al., 2014) C_T_ value of the three TcRefs as ΔC_T_ = (C_T•GOI_) − (C_T•*Tc*Refs_). Expression levels for each cacao gene in each sample were calculated as % cacao Ref = 100^*^[(E)^ΔCT^] where E equals the primer efficiency.

### Transcript profile analysis by machine learning

Tabulated raw counts of reads to each CDS were obtained from the bowtie alignment. Read counts were normalized using the DEseq package (Anders, 2010) and R x64 2.15.2 program (http://www.r-project.org/). Normalized RNA-Seq count data were log_10_ transformed and neural network self-organizing map (SOM) analysis (Kohonen, 1982, 1990) was employed (using MATLAB Neural Network Toolbox™) to objectively provide an unsupervised multi-variate and non-linear classification of the transcriptome into 24 different classes. The number of classes was chosen based on the number of treatments (control, Pmeg, and Ppal infection), number of samples, and allowing for differences in gene expression levels. The classification was done using a multivariate input vector describing the transcription response of each gene. GenAlEx 6.5 was then used to perform Principal Coordinates Analysis (PCoA) based on the covariance matrix with data standardization of log_10_ transformed RNA-Seq count data. Results from 24 of the most highly expressed genes from each of the 24 SOM classes were used to generate a 3-D scatter plot with an Excel macro add-in.

### Similarity between the criollo and forastero cacao genome

To establish the similarity between the two publicly available cacao genomes, the Criollo cacao genome (Argout et al., 2010) was subjected to bidirectional BLAST using the BLASTn search against the Forastero cacao genome (Motamayor et al., 2013).

### Statistical analysis

The data sets for the area under necrosis progression and pathogen load were normally distributed, as determined using the Shapiro–Wilk Test (Shapiro and Wilk, 1965) within the Statistical Package for the Social Sciences (IBM SPSS Statistics 22). The homogeneity of data sets across replicate experiments was confirmed within SPSS by two-tailed correlation analysis using mean data values (Pearson product moment; *r* ≥ 0.81; *P* = 0.01; Snedecor and Cochran, 1980). Therefore, data sets from the replicate experiments were pooled for the purposes of further statistical analysis. The significance of treatment effects was analyzed within SPSS by one-way ANOVA with *Post-Hoc* pair wise Least Significance Difference (LSD) comparisons (*P* = 0.05; Snedecor and Cochran, 1980).

Fisher's Exact Test between the four categories of genes, either up or down regulated (≥2-fold) for each species was carried out using the program Blast2GO (Conesa et al., 2005).

Relative expression data of cacao gene expression as % TcRefs was LOG-transformed to linearize the data (Rieu and Powers, 2009). Two-way RM ANOVA with Tukey's multiple comparisons test (*P* = 0.05) was carried out using GraphPad Prism version 7.0. A heat map of relative expression of the individual cacao genes was created using CIMminer (http://discover.nci.nih.gov/cimminer).

## Results

### Zoospore inoculated unwounded pod husk assay and secondary inoculum production

**Figure 1:**
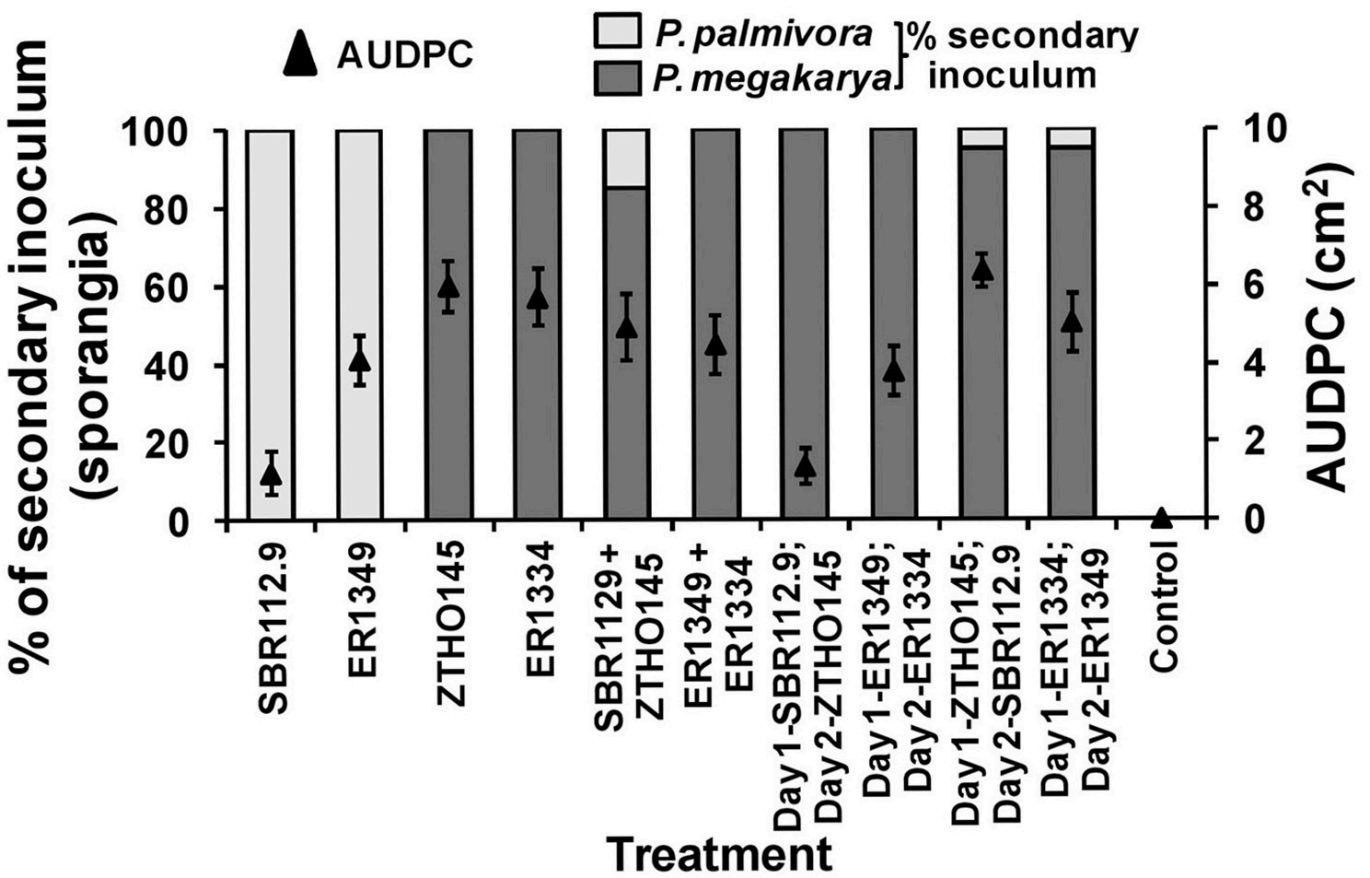
**Virulence responses and secondary inoculum production by *Phytophthora megakarya* and *P. palmivora* isolates as assessed in the zoospore inoculated unwounded pod husk assay**. Necrosis progression was observed up to 4 days and the area under disease progress curve (AUDPC) was calculated as described by Shaner and Finney (1977). The secondary inoculum loads were estimated based on the surface sporangia count of the infected pod husk pieces. Each experiment was repeated independently twice with three replicating husk pieces per isolate per experiment. Bars indicate SEM (LSD_0.05_ = 2.127%).

When zoospores of Pmeg and Ppal were applied to unwounded surfaces of cacao pod husks, the disease development, as measured by AUDPC, was dependent on the timing of the individual species isolate inoculated (Figure 1). When applied alone, the two Ppal isolates (IC-SBR112.9 and Gh-ER1349) had lower AUDPC values than the 2 Pmeg isolates (Ca-ZTHO145 and Gh-ER1334). When the Ppal zoospores were simultaneously inoculated along with Pmeg zoospores, the observed disease reactions were that of Pmeg (higher AUDPC values) and Pmeg was most often recovered from the sporangia produced at 5 dpi. When Pmeg zoospores were applied 24 h before Ppal zoospores, Pmeg was again recovered and the disease reaction was again that of Pmeg. Even when Ppal zoospores were applied to pod pieces 24 h before the Pmeg, Pmeg sporangia were recovered but the disease reactions were similar to that of the Ppal isolate included (a lower AUDPC resulted).

### Zoospore inoculated wounded pod husk assay and secondary inoculum production

**Figure 2:**
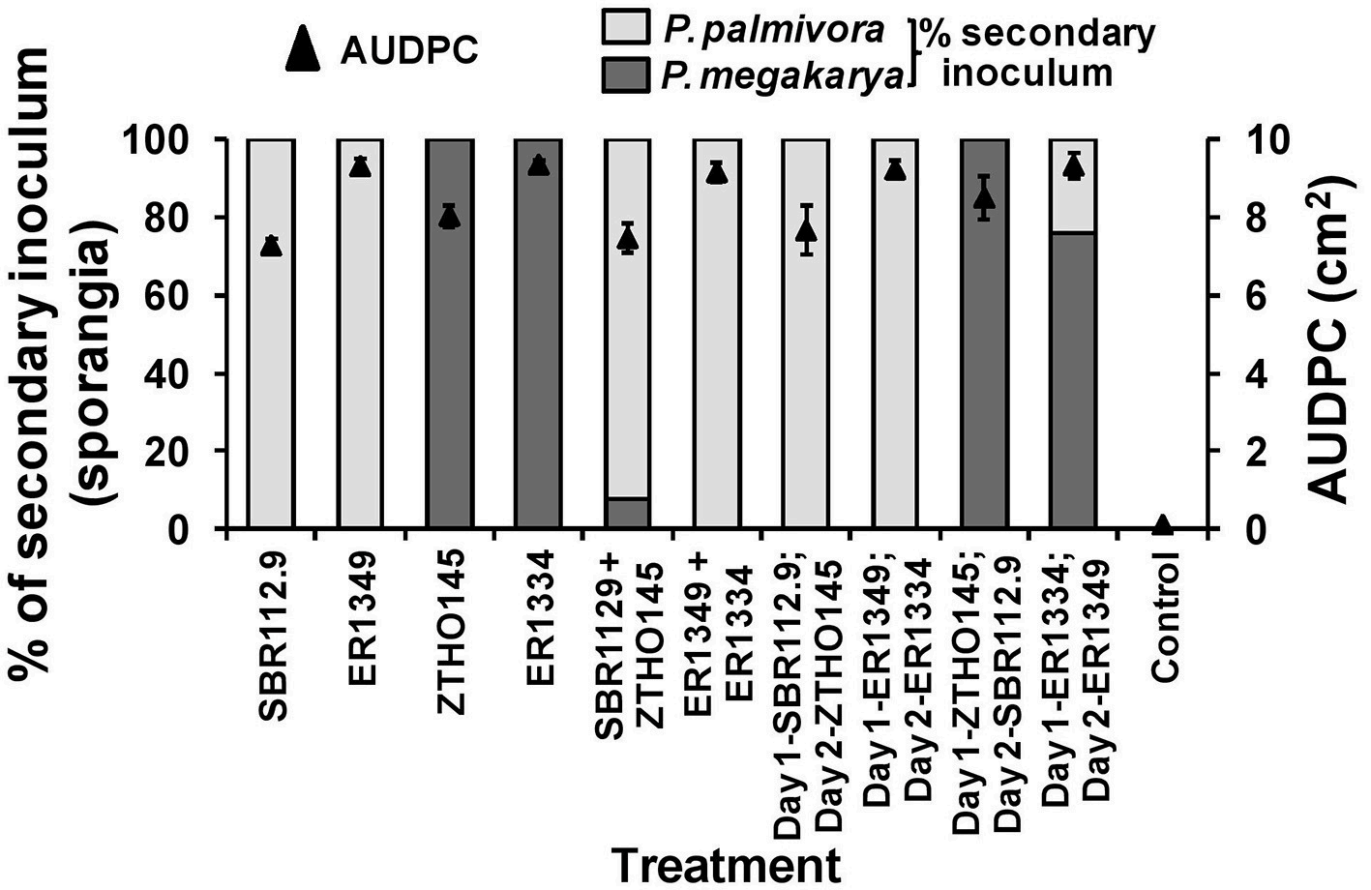
**Virulence responses and secondary inoculum production by *Phytophthora megakarya* and *P. palmivora* isolates as assessed in the zoospore inoculated wounded pod husk assay**. Necrosis progression was observed up to 4 days and the area under disease progress curve (AUDPC) was calculated as described by Shaner and Finney (1977). The secondary inoculum loads were estimated based on the surface sporangia count of the infected pod husk pieces. Each experiment was repeated independently twice with three replicating husk pieces per isolate per experiment. Bars indicate SEM (LSD_0.05_ = 1.19%).

When zoospores of Ppal (IC-SBR112.9 and Gh-ER1349) and Pmeg (Ca-ZTHO145 and Gh-ER1334) were applied to wounded surfaces of cacao pod husk pieces, the disease development (AUDPC) and species specific recovery of sporangia was again dependent on the timing of the individual species isolates inoculated but results differed compared to those observed for pod pieces with unwounded surfaces (Figure 2). When applied alone, the Ppal isolates had AUDPC values similar to that of the Pmeg isolates and higher than observed on unwounded surfaces. When Ppal zoospores were co-inoculated with Pmeg zoospores onto wounded surfaces, Ppal was most often recovered from the sporangia produced. When Ppal zoospores were applied 24 h before the Pmeg zoospores were applied, again Ppal was most often recovered. When Pmeg zoospores were applied 24 h before Ppal zoospores, Pmeg was recovered.

### Pathogen load in the infected tissue

**Figure 3:**
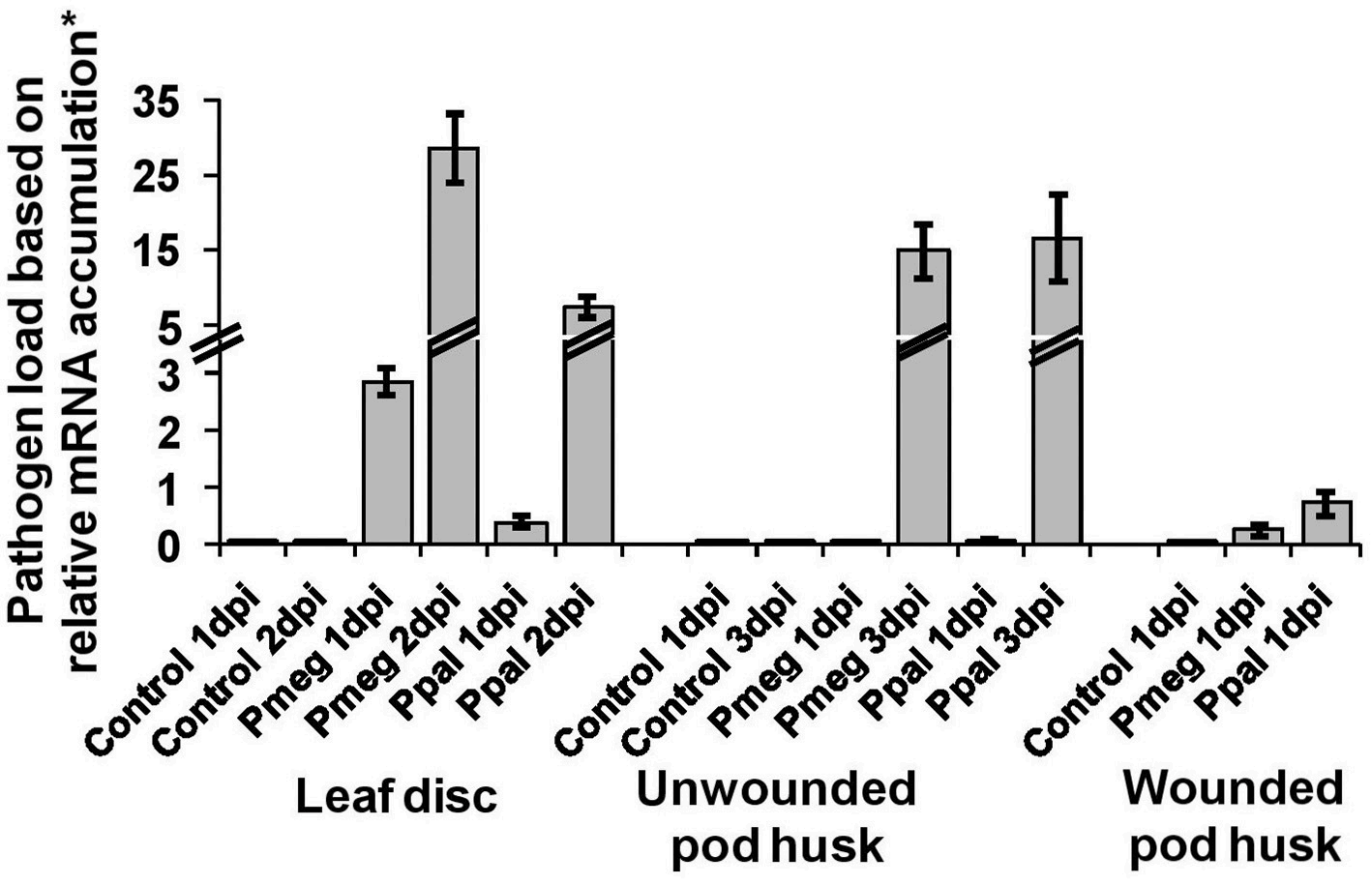
**Pathogen load based on relative expression of *Phytophthora* reference gene expression**. The expression level of the *P. megakarya* and *P. palmivora* reference genes (*Pm*TP/*Pp*TP) in each sample was calculated as % of the three cacao reference genes [100^*^(E) ^ΔCT^] where E equals the primer efficiency. The pathogen load was estimated as the geometric mean of the expression levels. Bars indicate SEM [LSD_0.05_(leaf disc) = 0.94; LSD_0.05_(Uw pod) = 1.2; LSD_0.05_(W pod) = 3.2].

Pathogen load was estimated as the percent of the expression levels of Pmeg/Ppal reference genes (*Pm*TP/*Pp*TP) relative to the expression of three cacao reference genes (detailed in experimental procedures). Pathogen load showed significant temporal variation for all three tissue types, leaf disks, wounded pod, and unwounded pod husk (Figure 3). There was a significant difference between Pmeg and Ppal load in infected leaf discs (*p* < 0.05) with Pmeg biomass being higher at both 1 and 2 dpi, but there was no significant difference in Pmeg and Ppal biomass (*p* > 0.05) in wounded or unwounded pod husks (Figure 3).

### RNA-seq analysis

The RNA-Seq analysis approach used Pmeg (isolate Gh-ER1334) and Ppal (isolate Gh-ER1349) infected pod husk samples (3 dpi) from the susceptible clone Catongo. The pathogen loads were around 15% (% of *PmTP*/*PpTP* expression relative to TcRefs expression), a level of pathogen expected to give reasonable detection of Pmeg/Ppal transcripts based on previous experiments (Ali et al., in press). The RNA-Seq analysis identified 19,022, 19,392, and 19,393 possible cacao transcripts (with >10 RPKM) for the libraries of untreated control, Pmeg and Ppal infected samples respectively (see Supporting Excel File S2, Sheet A). After normalization, the base mean number of reads for each cacao reference gene varied among samples as follows: *TcACP1* (4,459–4,847 reads), *TcGAPDH* (2,104–3,980 reads), and *TcACT* (63,879–80,794 reads). The geometric mean range for the three cacao reference genes as calculated for each clone varied from 8,573 to 11,227 reads, a difference of 23% or less between treatments after normalization.

There were 4,482 and 5,264 differentially expressed (>2-fold) transcripts between untreated control and Pmeg and Ppal infected samples, respectively, based on a *p* ≤ 0.05 (Benjamini and Hochberg, 1995). There were 3,181 and 3,521 transcripts induced and 1,301 and 1,743 transcripts repressed in Pmeg and Ppal compared to the untreated control, respectively (see Supporting Excel File S2, Sheet A). There were 463 transcripts induced and 580 transcripts repressed in response to Pmeg infection compared to Ppal infection although in most instances the trends were the same for both species with only the relative fold change varying. Among all the differential expression transcripts, 3,898 transcripts encode proteins with putative functions. For a better identification of the transcripts we established the similarity between two publicly available cacao genomes (Argout et al., 2010; Motamayor et al., 2013). There are 38,609 homologous genes (*E* < 1e-10) shared between the Criollo and Forastero cacao genomes (see Supporting Excel File S2, Sheet B).

### Self-organizing map (SOM) analysis

**Figure 4:**
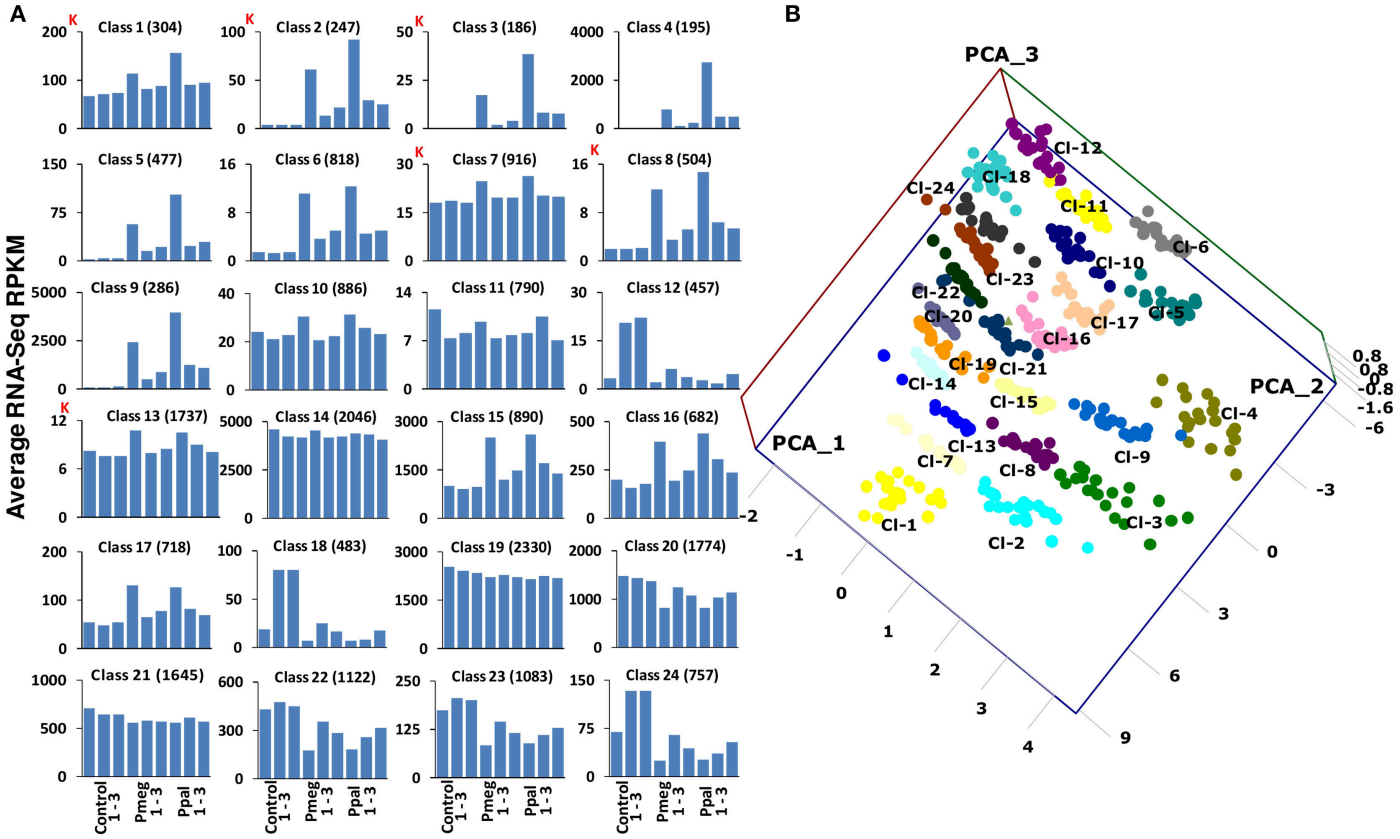
**Self-organizing map (SOM) classification of *Phytophthora megakarya* and *P. palmivora* affected cacao genes based on the RNA-Seq count data (3 dpi) and their relationship. (A)** Using machine learning techniques cacao genes were classified into 24 different multi-variate and non-linear SOM classes. Average RNA-Seq counts (RPKM) for each library across the 24 classes indicates the gene expression pattern (K indicates 1,000 counts). **(B)** Principal Coordinate Analysis (PCoA) of the log_10_ transformed RNA-Seq count data of the 24 most highly expressed gene models from the 24 SOM classes, plotted on a 3D scatter plot.

To compare global cacao gene expression profiles in response to the two *Phytophthora* species, we used neural network software to produce SOM (Kohonen, 1982, 1990) from the RNA-Seq data. This enabled us to identify common patterns of gene expression in response to Pmeg/Ppal infection without matching genes one-to-one between treatments. Cacao transcripts (with >10 RPKM) for the libraries of untreated control, Pmeg, and Ppal infected samples were classified into 24 multi-variate and non-linear SOM classes (Figure 4A and see Supporting Excel File S1, Sheet B). Among the 24 SOM classes generated, classes 1, 2, 3, 7, 8, 13, 14, and 19 were highly expressed of which, classes 14 and 19 largely showed constitutive expression across all the RNA-Seq libraries with expression level being the primary divider. More than 80% of the genes belonging to classes 2, 3, 4, 5, 6, 8, and 9 were significantly induced in response to Pmeg/Ppal infection, whereas, between 20 and 30% of the genes belonging to classes 1, 6, 15, 16, and 17 were also significantly induced in response to Pmeg/Ppal infection (see Supporting Excel file S1, Sheet B). Genes repressed in response to Pmeg/Ppal infection were mostly grouped in class 12, 18, 22, 23, and 24. PCoA was carried out using log_10_ transformed RNA-Seq count data of the 20 most highly expressed GeneMs from each of the 24 SOM classes. A 3D scatter plot PCoA comparison of coordinates 1, 2, and 3 is presented (Figure 4B). These coordinates account for 54.46, 15.37, and 11.26% of the data variation, respectively. As expected, genes from each of the SOM classes clustered separately, according to infection specificity and transcript level.

### GO and KEGG analysis

**Figure 5:**
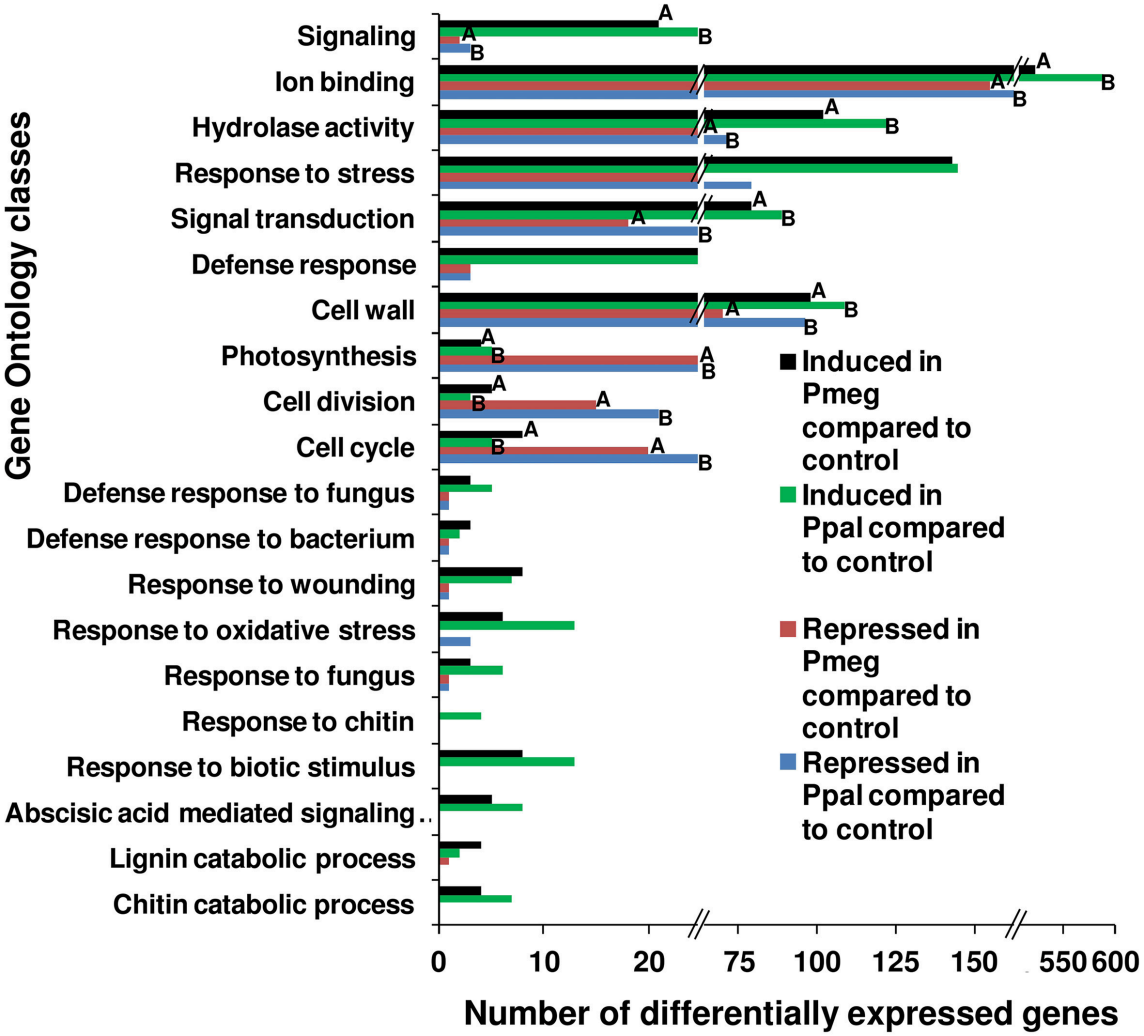
**Shift in the number of differentially expressed genes among key biological processes involved in the disease response in *Phytophthora megakarya* and *P. palmivora* infected cacao pod husk samples**. Differentially expressed genes were identified using RNA-Seq analysis between control and infected pod husk samples (at 3 dpi) and grouped into four categories, either up or down regulated (≥2-fold) for each species. Gene ontology (GO) analysis was carried out using the program Blast2GO (Conesa et al., 2005) comparing up and down regulated categories within species and up regulated categories and down regulated categories between species. Within GO classes, bars with same letters are significantly different (FDR < 0.05 along with *P* < 0.05) as estimated by Fisher's Exact Test within the Blast2GO suite. Where letters are not indicated, no differences were observed. Underlying data are shown in Supplementary Excel File S1, Sheet C and D.

Gene ontology (GO) analysis using Fisher's Exact Test of the 4 categories of differentially expressed genes (>2-fold) responding to Pmeg/Ppal showed substantial differences (Figure 5 and Supporting Excel File S1, Sheet C). Genes belonging to GO classes corresponding to signaling, ion binding, hydrolase activity, cell wall, and signal transduction were more commonly observed in the induced categories than in repressed categories (FDR < 0.05 along with *P* < 0.05) in response to Pmeg/Ppal infection. Whereas, genes belonging to GO classes photosynthesis, cell division, and cell cycle were more commonly observed in the repressed categories than in the induced categories (FDR < 0.05 along with *P* < 0.05) in response to Pmeg/Ppal infection (Figure 5). Often genes belonging to defense associated GO classes (for example defense response, response to wounding, oxidative stress and biotic stimulus) were induced in response to Pmeg/Ppal infection but their GO classes did not meet the required level of significance (Figure 5). Most importantly, the Fisher's Exact Test in the blast2go suite indicated there were no differences for any GO class when directly comparing cacao gene categories induced by Pmeg/Ppal infection or repressed by Pmeg/Ppal infection (Figure 5 and Supporting Excel File S1, Sheet D).

Initially KEGG analysis was carried out on genes with the 8 highly expressed SOM classes (SOM class 1, 2, 3, 7, 8, 13, 14, and 19; see Figure 4) previously identified. Through this processes, candidate genes were identified for members of many pathways, often completing or nearly completing the pathways (see Supporting Excel File S1, Sheet E). Examples of particular relevance to this study include key pathways involved in the plant defense response including alpha-linolenic acid metabolism (jasmonate biosynthesis; ko00592), cysteine and methionine metabolism (ethylene biosynthesis; ko00270), plant-pathogen interaction (ko04626), endocytosis (ko04144), plant hormone signal transduction (ko04075), plant secondary metabolites including phenylpropanoid biosynthesis (ko00940), phenylalanine, tyrosine, and tryptophan biosynthesis (ko00400), and terpenoid backbone biosynthesis (ko00900). KEGG pathways analysis was then carried out incorporating 4 individual differentially expressed gene sets (>2-fold up or down regulated comparing Pmeg/Ppal infected to control), identifying 149 different metabolic pathways (see Supporting Excel File S1, Sheet F). The top 10 KEGG pathways containing the largest numbers of induced genes in response to Pmeg/Ppal infection were biosynthesis of amino acids, carbon metabolism, plant hormone signal transduction, starch and sucrose metabolism, amino sugar and nucleotide sugar metabolism, endocytosis, plant-pathogen interaction, glycolysis/gluconeogenesis, protein processing in endoplasmic reticulum and carbon fixation in photosynthetic organisms (see Supporting Excel File S1, Sheet F). The top 10 KEGG pathways containing the largest numbers of repressed genes in response to Pmeg/Ppal infection were photosynthesis, cell cycle, starch and sucrose metabolism, carbon metabolism, biosynthesis of amino acids, plant hormone signal transduction, photosynthesis—antenna proteins, porphyrin, and chlorophyll metabolism, DNA replication and carbon fixation in photosynthetic organisms (see Supporting Excel File S1, Sheet F). Since both Pmeg and Ppal infection showed induction and repression of similar pathways, we carried out KEGG pathways analysis of 2 additional gene sets, induced by either or both Pmeg and Ppal infection and repressed by either or both Pmeg and Ppal infection (see Supporting Excel File S1, Sheet G). As expected, shifts in expression were observed in key pathways involved in the plant defense response mentioned above. There were 16 KEGG pathway ko04626 (plant-pathogen interaction) gene members showing more than two-fold induction in response to Pmeg/Ppal in infection (See Supplementary Table S1). The cacao genes induced include putative pathway recognition/signal components chitin elicitor receptor kinase 1 (CERK1), cyclic nucleotide gated channel (CNGC), and LRR receptor-like serine/threonine-protein kinase FLS2 (FLS2). In addition, at least one putative BAK1 (brassinosteroid insensitive 1-associated receptor kinase 1) candidate gene showed induction although less than the two-fold threshold used prior to KEGG analysis. Seventeen gene members associated with endocytosis (ko04144) showed up-regulation, with 7 of these being associated with late endosomes (See Supplementary Table S2). In addition, the pathway leading to jasmonic acid (JA) biosynthesis (ko00592 alpha-Linolenic acid metabolism) has 11 pathway steps with genes represented as induced in response to Pmeg/Ppal infection, again a near complete pathway (See Supplementary Table S1). Twenty two candidate members of KEGG pathway ko04075 (plant hormone signal transduction) showed induction in response to Pmeg/Ppal infection (See Supplementary Table S1). Multiple members of four primary genes in the ethylene biosynthesis pathway (within the cysteine and methionine metabolism pathway ko00270) were also induced (See Supplementary Table S1). Plant secondary metabolites including 12 candidate members of KEGG pathway ko00400 (phenylalanine, tyrosine, and tryptophan biosynthesis) showed induction in response to Pmeg/Ppal infection; 8 members of the terpenoid backbone biosynthesis pathway (ko00400) showed induction of two-fold or more with 2 additional members showing induction but less than two-fold and 8 genes in pathway terpenoid backbone biosynthesis (ko00900) showing induction (See Supplementary Table S3).

### Expression of genes encoding pathogenesis related proteins (Pr-proteins)

At least 240 genes putatively encoding Pr-related proteins were considered expressed (Table 1, mean expression ≥10 reads for at least one treatment) in the RNA-Seq analysis representing fourteen of the 17 characterized classes of Pr-proteins (Table 1). Infection repressed expression of 47 and 54 genes encoding Pr-related protein for Pmeg and Ppal, respectively while 81 and 87 genes encoding Pr-related proteins were induced by Pmeg and Ppal infection, respectively. Only 10 genes encoding Pr-related proteins were more highly expressed in response to Pmeg infection compared to Ppal infection while 24 genes encoding Pr-related proteins were more highly expressed in response to Ppal infection compared to Pmeg infection.

**Table 1:** **Summary of expression of genes encoding putative Pr-proteins in cacao pod pieces responding to infection by Pmeg and Ppal as determined by RNA-Seq analysis (See supporting Excel File S2, Sheet A)**.

**Pr-protein family**	**Total**	**No. expressed**	**Down-regulated**		**Up-regulated**		**Differentially expressed**		**Compared to (Fister et al**., **2016****)**	
			**Pmeg**	**Ppal**	**Pmeg**	**Ppal**	**Pmeg>Ppal**	**Ppal>Pmeg**	**Similar**	**Different**
Pr-1	14	10	1	1	4	6	1	0	1	0
Pr-2	43	31	10	16	8	8	1	1	5	2
Pr-3	11	11	1	1	6	6	0	3	6	2
Pr-4	8	5	2	2	1	1	0	0	0	3
Pr-5	30	20	5	5	6	7	0	1	3	3
Pr-6	8	7	2	1	5	5	0	0	4	1
Pr-7	54	34	14	16	6	7	2	2	3	2
Pr-8	14	9	0	0	3	2	0	0	1	1
Pr-9	81	59	10	11	15	13	6	6	4	9
Pr-10	23	16	1	0	13	15	0	1	8	0
Pr-11	11	10	0	0	3	5	0	3	1	2
Pr-12	3	0	0	0	0	0	0	0	0	0
Pr-13	0	0	0	0	0	0	0	0	0	0
Pr-14	16	4	0	0	0	0	0	0	0	2
Pr-15	0	0	0	0	0	0	0	0	0	0
Pr-16	38	21	1	1	8	9	0	7	5	3
Pr-17	5	3	0	0	3	3	0	0	2	1
Total	359	240	47	54	81	87	10	24	43	31

*For inclusion a minimum of 10 RNA-Seq RPKM for at least one treatment mean (control, Pmeg, and Ppal) and a 2-fold induction or repression due to Pmeg/Ppal infection was required. Expression patterns were also compared to those shown differentially expressed in cacao leaves responding to Ppal infection (Fister et al., 2016)*.

### RNA-seq validation and temporal and tissue specific expression analysis of cacao genes using RT-qPCR

**Figure 6:**
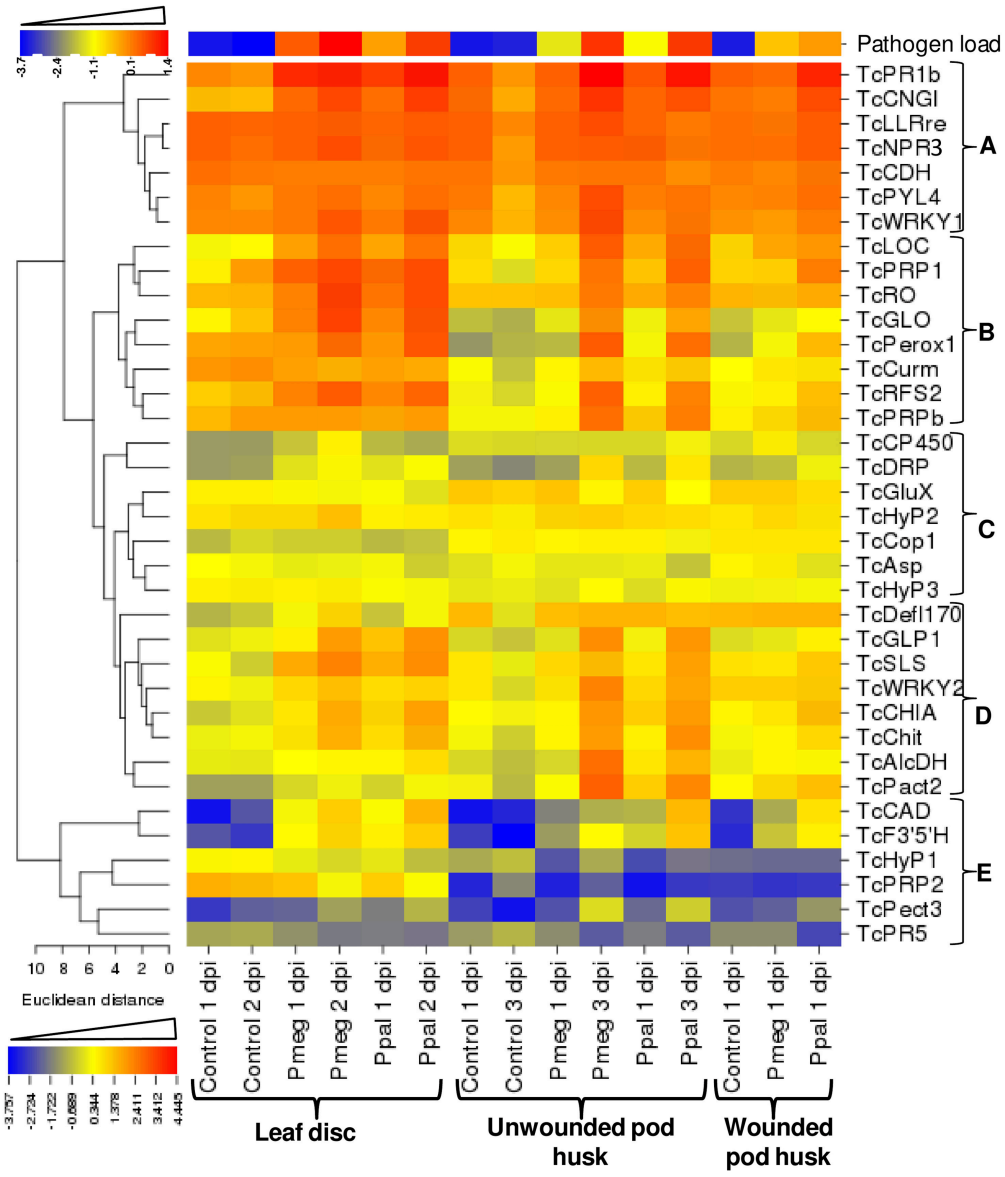
**Relative expression of *Phytophthora* reference genes and 36 *Theobroma cacao* genes expression analyzed over *Phytophthora megakarya* and *P. palmivora* infected and control samples of cacao leaf disc, wounded, and unwounded pod husk**. Relative mRNA expression levels were quantified relative to that of the three cacao reference genes (geometric mean) by the E^−ΔΔCt^ method (Livark and Schmittgen, 2001), where E equals the primer efficiency. The relative mRNA expression levels were LOG_10_-transformed to linearize the data, then the heat map was created using CIMminer (http://discover.nci.nih.gov/cimminer).

Using the 9 RNA samples used for RNA-Seq analysis, 17 out of the 19 cacao genes selected to show varied reactions to treatments had correlation coefficients >0.80 between the RNA-Seq and RT-qPCR expression results (13 with *p* ≤ 0.05) (see Supporting Excel File S1, Sheet H). In addition to these original 19 genes, another set of 17 cacao genes were selected based on their involvement in specific pathways including plant hormone signal transduction and plant-pathogen interactions and analyzed for temporal and tissue specific expression in response to Pmeg/Ppal infection (Figure 6 and see Supporting Excel File S1, Sheet I). Only 4 genes failed to show differential expression in response to *Phytophthora* infection in at least one time point/tissue combination (TcHyp2, TcHyp3, TcCop1, and TcCHD). Based on the euclidean distance of expression profile, the 36 genes can be categorized into 5 groups (Figure 6). Genes in group A were highly expressed in all the infected samples. These genes were repressed at the later time point in the control samples but showed steady or induced expression in infected samples. Genes in group B were highly induced in response to level of infection based on the pathogen load (Figure 6). Genes in group D were also induced in response to level of infection, but the level of induction was higher in infected pod husk (unwounded) compared to infected leaf disc. Genes in group C were either not differentially expressed or expression was altered in response to infection at a low level, and Group E genes were induced from very low constitutive expression levels (Figure 6 and see Supporting Excel File S1, Sheet I). Most of the genes studied showed coordinated regulation in response to infection by both *Phytophthora* species although in some cases the level and timing of induction was dependent on the *Phytopthora* species used (Figure 6 and see Supporting Excel File S1, Sheet I). The biggest difference was the more intense changes in expression for 14 of the 36 genes studied in Ppal inoculated wounded pod pieces.

**Figure 7:**
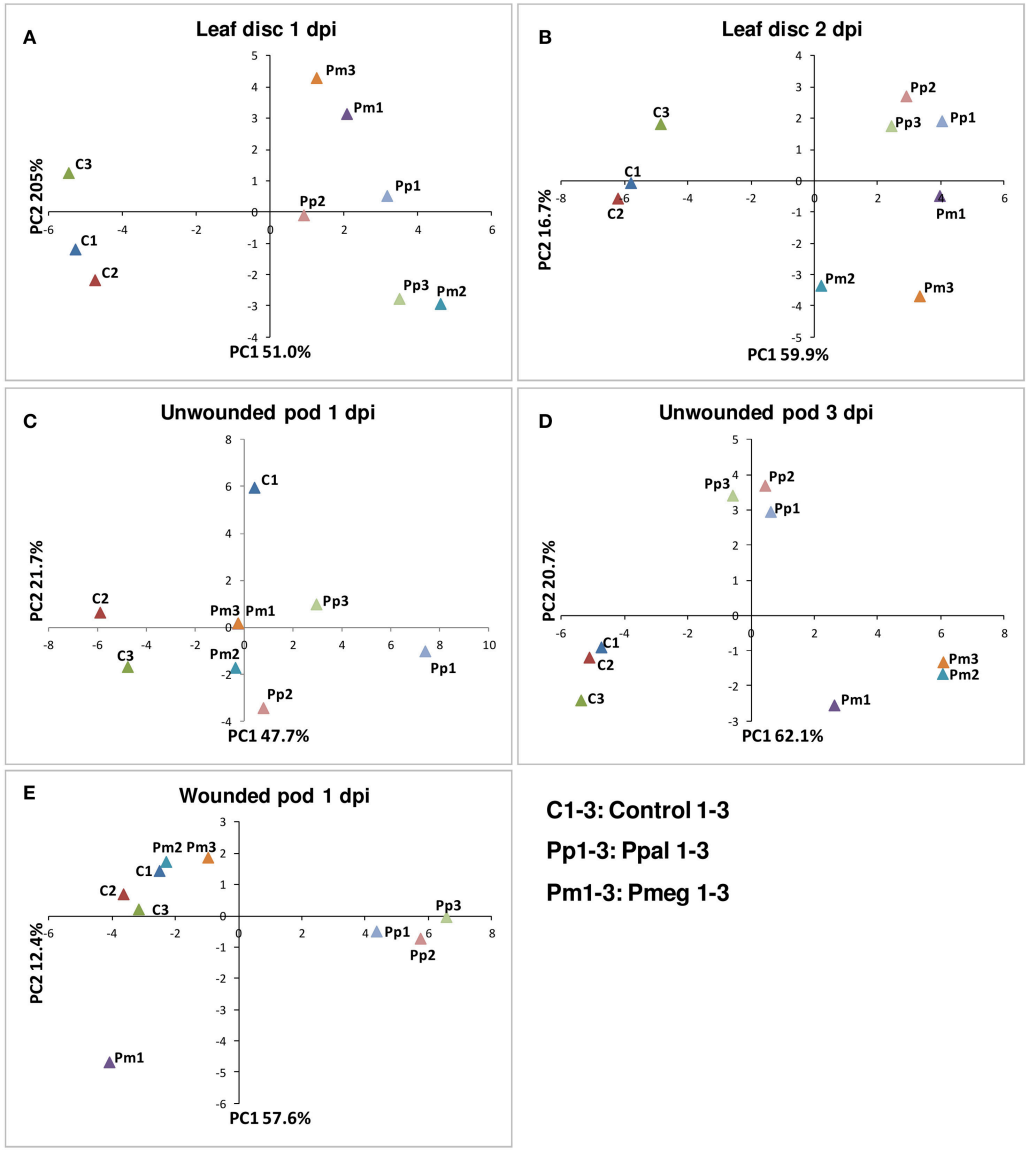
**Principal-components plot of the relative mRNA expression of the 36 genes selected based on RNA-Seq analysis**. **(A)** Leaf disc at 1 day post inoculation (dpi). **(B)** Leaf disc at 2 dpi. **(C)** Unwounded pod husk at 1 dpi. **(D)** Unwounded pod husk at 3 dpi. **(E)** Wounded pod husk at 1 dpi. RT-qPCR was conducted using RNA harvested from uninfected control, *Phytophthora megakarya* and *P. palmivora* infected samples.

PCoA was carried out using the RT-qPCR data from 36 genes for the 5 different treatment conditions (Figure 7). For each PCoA, the comparison of coordinates 1 and 2 is presented. In case of the leaf disc, a high amount of pathogen load was detected at 1 dpi and thus uninfected samples clustered separately from infected samples (Figure 7A). At the later time point (2 dpi), Ppal and Pmeg samples were further separated into upper and lower right quadrants (Figure 7B). In the case of unwounded pod husk, the level of infection was lower at 1 dpi and there was less separation of the samples between treatments but the Ppal treated unwounded pod husk samples were further separated from controls than the Pmeg samples (Figure 7C). At 3 dpi the Pmeg, Ppal and control samples clearly clustered separately in unwounded pod husk samples (Figure 7D). In wounded pod husks, only the Ppal treated samples clustered separately from control samples (Figure 7E).

## Discussion

Resistance to *Phytophthora* in cacao has the unusual characteristic in that resistance to one species has been correlated, through multiple studies, with resistance to all species (Bailey et al., 2015). This means, that although Pmeg is the most aggressive species on cacao, cacao genotypes rank similarly in their reactions to Pmeg as they do to other *Phytophthora* species. It implies that the cacao preformed and induced defense mechanism function against *Phytophthora* regardless of the species. In a meta-analysis of all QTLs for resistance to BPR, 48 QTLs were reduced to 13 consensus QTLs (Lanaud et al., 2009) lending support to the general hypothesis that resistance to BPR is quantitative and that multiple genetic sources of resistance exist. The existence of specific R-genes that mediate qualitative resistance has not been demonstrated. If it is true that resistance to one species of *Phytophthora* correlates to resistance to all *Phytophthora* species, it is possible non-interaction dependent differences between Pmeg/Ppal species or varying abilities between the species to degrade or bypass cacao defense mechanisms may account for these differences. For example the ability to form appressoria and sporangia vary between Pmeg and Ppal (Ali et al., 2016). In this study, susceptible interactions between Pmeg/Ppal and *T. cacao* are considered but since susceptibility/resistance is relative and incomplete, aspects of plant defense in these interactions might still be found.

The four isolates used in the studies using Pmeg/Ppal mixed inoculums gave similar individual reactions in an earlier study to those seen here (Ali et al., 2016). In that study, Ppal displayed a wide divergence in disease reactions with many isolates, including IC-SBR112.9, showing low virulence while Pmeg isolates were more uniform in virulence. When Pmeg and Ppal were co-applied to an unwounded pod surface, predominantly Pmeg sporangia were recovered regardless of the order of application. Pmeg has been shown to produces appressoria more often than Ppal allowing direct penetration whereas Ppal more commonly penetrated through stomata (Ali et al., 2016). This alone could account for the ability of Pmeg to dominate the infection court when simultaneously applied. Surprisingly, even when Ppal was applied to the infection court 24 h before Pmeg, Pmeg still dominated in the production of secondary inoculums. It has also been noted that Pmeg sporulates more quickly within the expanding zone of necrosis than Ppal on infected pods (Bailey et al., 2015), again giving it an advantage. These results support the observations that Pmeg is more aggressive than Ppal in causing disease on unwounded cacao tissues (Ali et al., 2016) and has implications as to why Pmeg has displaced Ppal from cacao in countries like Nigeria and Cameroon (Nyasse et al., 1999; Ndubuaku and Asogwa, 2006; Djocgoue et al., 2007). Although, the disease reactions (AUDPC) to co-inoculations resemble that of Pmeg when Pmeg zoospores are applied earlier or simultaneously with Ppal zoospores, when Ppal zoospores are applied first the disease reaction are limited resembling that of the Ppal isolate used. This suggests Ppal infections may limit secondary infection by Pmeg at some level perhaps triggering a stronger plant defense response.

In sharp contrast to the unwounded system, Ppal dominates the infection court when simultaneously applied to a wounded pod surface producing secondary inoculum in preference to Pmeg. Lesions from Ppal tend to expand at a faster rate than those of Pmeg when infections are through wounds (Brasier and Griffin, 1979). Again, as observed in earlier studies (Ali et al., 2016), Ppal benefited from a wounded surface differentially compared to Pmeg with less virulent Ppal isolates causing more disease compared to the reactions on unwounded surfaces. In fact, both species caused accelerated disease when infecting through wounds. Any benefit Pmeg might have due to direct penetration by appressoria is negated by wounding. Wounding, whether using mycelia or zoospores for inoculum, may also bypass/alter the biotrophic phase. Ppal benefitted from a wounded surface but Pmeg was not inhibited by a wounded surface. When Pmeg was applied to the wound site first it dominated the infection court. When applied together, Ppal and Pmeg dominated the infection court under different circumstances (Pmeg without wounding and Ppal with wounding). The observed differences occurred despite a close similarity in the plant's susceptible reactions to their individual infections as shown by RNA-Seq analysis. This supports the contention that specific aspects of the two species' biology, for example Pmeg's ability to survive a dry cycle (Bailey et al., 2015) and directly infect using appresoria (Ali et al., 2016) and Ppal's ability to exploit available nutrients (Zoberi et al., 1981; Ali et al., 2016), are important in determining which species dominates in the field rather than simply their individual abilities to alter/suppress the plant defense responses.

Though differential transcript accumulation does not prove that a gene is directly involved in Pmeg/Ppal tolerance or susceptibility, many of the transcriptional differences detected were related to pathways and processes associated with plant defense. The RT-qPCR study of the 36 genes clearly validated the RNA-Seq findings, the induction of these genes by Pmeg/Ppal infection and the association of expression with pathogen load, suggests that they are likely involved in the general plant defense and stress responses. The significant positive correlation among these genes and between their RNA-Seq and RT-qPCR results indicates collective coordination of their expression in response to Pmeg and Ppal infection.

As the plant reaction studied here was part of a susceptible interaction between cacao and Pmeg/Ppal, GO analysis of four differentially expressed cacao gene categories assembled from the RNA-Seq result likely identifies differentially induced and repressed GO classes associated with the susceptible interaction between plant and pathogen (Ali et al., 2014; Tan et al., 2015). The absence of any GO class showing significant difference between Pmeg and Ppal infection within the induced and repressed categories further indicates that the susceptible interactions between cacao and the two species are very similar. RT-qPCR analysis of the 36 genes showed distinct variations in expression patterns based on temporal and tissue specificity, which were related to pathogen load. The expression patterns were again generally similar between Pmeg and Ppal infected pod husk and leaf samples but some differences were observed (Figure 6 and see Supporting Excel File S1, Sheet I). Ppal tended to induce changes in gene expression earlier (at 1 dpi) in the unwounded pod system and in wounded pods Ppal induced stronger reactions at 1 dpi for 14 of the 36 genes studied. PCoA of the relative mRNA expression of the 36 the RT-qPCR tested genes shows that global gene expression patterns differ between Pmeg and Ppal infected tissue as the infection progresses. As tendencies in the RNA-Seq and the wounded and unwounded pod studies were similar, the suggestion is cacao defense responses are more actively induced against Ppal or possibly Pmeg is better able to suppress these reactions. Similar studies focused on earlier time points and incorporating tolerant clones should help clarify this point. Although, the constitutive expression levels detected in leaf disc vs. pods often varied, leaf discs and pod pieces generally responded the same to infection by Pmeg/Ppal showing similar tendencies toward induction/repression for the 36 genes studied using RT-qPCR. The leaf assay, as used to measure resistance to Pmeg and its correlation to pod resistance, has been the topic of much discussion in the cacao research community. Iwaro et al. (1997a) found that, although leaf and pod resistance at the post penetration levels were highly correlated, they were not correlated between clones at the penetration level. It was noted that stomatal frequencies were not highly correlated between leaves and pods, possibly explaining the disconnection when measuring penetration resistance. Stomatal development is incomplete in young leaves like those used here (deAlmeida and Valle, 2007) and information on stomatal development in pods is difficult to obtain from the literature. The positive results linking resistance in cacao pods to resistance in cacao leaves seem to center around components potentially shared by leaves and pods, most notably phenol reactions, lignification processes, and wax layers (Ndoumou et al., 1995; Djocgoue et al., 2007; Nyadanu et al., 2012), aspects of resistance/tolerance that might be expected to show consistent reactions.

### SOM class analysis sorts genes based on expression level and response to *Phytophthora* infection

Conventional RNA-Seq data analysis mainly focuses on differential induction or fold change but, it is also important to also consider mRNA expression level. Machine learning approaches, such as unsupervised SOM classification, allow us to consider not only fold change and expression level, but other unaccounted for variables. SOM is an unsupervised classification technique that reduces the dimension of data through the use of self-organizing neural networks, not imposing *a priori* class assumptions, but allowing the data to determine how genes should be classified. SOM-based gene expression analyses have been applied in diverse studies including studies on cell differentiation and development, organogenesis and tumor differentiation (Wirth et al., 2011). In this study, in addition to differential gene expression in response to treatment, a wide range in relative expression levels were demonstrated. In many cases, genes with “significant” differential expression associated with treatments were expressed at very low levels. This is especially true for many of the genes down-regulated in response to *Phytophthora* infection (SOM classes 12, 18, and 24). Often within a pathway, genes showing differential expression shared similar expression levels along with similar expression patterns. Clearly not all plant defense genes need to be induced to function. In some cases, individual members of a pathway failed to show induction but instead showed high constitutive expression. Furthermore, genes grouped together by SOM-based expression analysis can be used to set priorities for promoter analysis and other gene comparisons based on their similar regulation.

### Expression of genes encoding Pr-protein is significantly altered in pods by Pmeg/Ppal infection

In a recent study using microarray technologies, Fister et al. (2016) identified 359 gene members within the cacao genome encompassing 15 of the 17 characterized classes of Pr-proteins. Approximately 50% of these genes are organized in tandem arrays within the cacao genome (Fister et al., 2016). Of the PR gene members, 67 were induced and 7 were repressed in cacao leaves in response to Ppal infection (Fister et al., 2016). In many cases leaves have been used as proxies due to the difficulty in acquiring pods in research settings. Since diseases like BPR primarily cause losses in yield by destroying the fruit directly, more studies focused on pods are needed. In the current study, all but 119 Pr-protein gene family members were found to be expressed (Table 1, RPKM ≥ 10 for at least one treatment) in pod which compares to the 126 gene members removed by the background filtration of the Fister et al. (2016) leaf microarray data set. Of the 67 Pr-protein gene family members induced in leaves infected by Ppal (Fister et al., 2016), 38 and 30 were also induced in cacao pod pieces inoculated with Ppal and Pmeg, respectively. In many cases, genes highly induced in leaves by Ppal were found to be expressed at high levels constitutively in control pod pieces and induced either at low levels or not at all in response to *Phytophthora* infection. Conversely, Pr-protein gene members induced at low levels in leaves often had low constitutive expression and were highly induced in pod pieces. On the surface this suggests basic differences in how genes encoding Pr-proteins respond to *Phytophthora* infection in pods and leaves but this would need to be verified using the same cacao germplasm. In total, 87 and 81 Pr-protein gene family members were induced in pod pieces inoculated with Ppal and Pmeg, respectively. In most cases, the genes observed uniquely induced in the current study also had low constitutive expression in pod pieces. Genes with high constitutive expression and limited induction tended to be placed in SOM class 1 whereas the genes with low constitutive expression and high induction tended to be placed in SOM classes 2 and 3. In our study, 54 and 47 Pr-protein genes were repressed in pod pieces responding to infection by Ppal and Pmeg, respectively, and these tended to fall into SOM classes 18, 22, 23, and 24. No genes encoding Pr-13 and 15 were found in cacao (Fister et al., 2016) and no genes encoding Pr-12 or Pr-14 were differentially expressed compared to control samples in response to *Phytophthora* infection.

### KEGG analysis identifies plant defense related pathways up-regulated in response to *Phytophthora* infection

Although, genes associated with many different KEGG pathways, some participating in basic aspects of pod physiology, were altered in response to *Phytophthora* infection, pathways associated with plant defense would seem to be most pertinent. For example, CERK1, CNGC, FLS2, and BAK1 modulate PAMP signal perception and transduction (Wu et al., 2014; Saand et al., 2015). Endocytosis is critical to the subsequent induction of plant innate immunity through FLS2 and BAK1 which participate together in this process (Robatzek et al., 2006; Chinchilla et al., 2007). FLS2 is targeted to the vacuole after PAMP recognition, phosphorylation, and ubiquination through the late endosome. Expression of several components associated with the FLS2 signaling pathway (or a parallel system in this case) was enhanced ending with the up-regulation of a constitutively highly expressed Pr-1 gene (Tc02_g002410). The most notable gene associated with endocytosis includes several Rab7 candidates, the encoded Rab7 protein being recruited to the extrahaustorial membrane (EHM) in interactions with *Phytophthora* (Bozkurt et al., 2015). BAK1 but not FLS2, is also recruited to the EHM (Bozkurt et al., 2015). Although, the inductions observed in the plant-pathogen interaction pathways were significant, the interactions studied herein were susceptible interactions. Moreover, the infected materials studied were in the necrotrophic phase. Therefore, the plant responses may have been limited compared to the reactions if immunity were induced.

Plant hormones are intimately intertwined with the induction of both susceptibility and resistance (Bari and Jones, 2009). Differential expression was noted for candidate genes associated with the actions of auxins, gibberellins, cytokinines, ABA, BRs, JA, and salicylic acid (SA). There were 14 candidate gene members associated with pathway ko04075 (plant hormone signal transduction) showing down-regulation and in many cases they represent orthologs of genes induced in the same pathway. Notably no members of the ethylene, JA or SA signal transduction pathways, pathways that are closely associated with plant defense, showed repression. The list of candidate genes showing induction in response to *Phytophthora* infection complete or nearly complete, respectively, the ethylene (Wang et al., 2002) and JA (Wasternack, 2007) biosynthesis pathways (See Supplementary Table S1). Though ethylene and JA play a major role in plant defense (Mersmann et al., 2010; Santino et al., 2013; Broekgaarden et al., 2015), induction of ethylene and JA related genes does not always mean involvement in resistance (Eshraghi et al., 2014).

From the literature, little support can be found for JA participating in penetration resistance to *Phytophtora* (a hemibiotroph) infection. Instead, SA is more often implicated (Halim et al., 2007; Loake and Grant, 2007). The SA signal transduction pathway was highlighted by the KEGG analysis. The non-expressor of PR1 (NPR1) plays a central role in regulating the transcriptional response to plant pathogens through the SA pathway (Fellbrich et al., 2002). NPR3, on the other hand, functions as a repressor of NPR1-mediated defense response (Shi et al., 2013a). Functional orthologs of NPR1 (Tc09_g007660) and NPR3 (Tc06_g011480) have been identified in cacao (Shi et al., 2010, 2013b) and the “NPR1” identified in our KEGG analysis as induced has been annotated and functionally proven to instead be NPR3 (Shi et al., 2013a). The functionally annotated NPR1 gene in cacao showed constitutive expression in the RNA-Seq analysis. NPR3's induction supports the contention that the Pr-1 gene induced in response to *Phytophthora* was more reasonably associated with the FLS2/BAK1signalling pathway (or rather a parallel system in the cacao/*Phytophthora* interaction). Along the same lines, although a candidate BAK1 gene showed up-regulation, a candidate BRI1 gene was down-regulated. This is of interest since BAK1 shares the ability to interact with FLS2 of the plant defense pathway and BRI1 of the brassinosteroid signaling pathway (Wang, 2012). In this case, the brassinosteroid signaling pathway appears more likely down regulated, something observed in a previous study of the interaction between cacao and *Moniliophthora roreri*, the causal agent of frosty pod rot (Ali et al., 2014).

Several component pathways associated with secondary metabolite production showed significant induction in response to *Phytophthora* infection. Although, genes identified were not always hugely induced or highly expressed, they make up almost the entire biosynthetic pathway from D-erythrose- 4-phosphate to phenylalanine. From phenylalanine, 13 members of the phenylpropanoid biosynthesis (PAL) pathway (ko00949) were induced, again largely completing the pathway. The PAL pathway is well studied and known to contribute to plant defense by its many off shoots including hydroxycinnamic acids, monolignols/lignin, flavonoids, isoflavonoids, and stilbenes (Dixon et al., 2002). A notable pathway gene member missing from this list is cinnamoyl-CoA reductase (EC:1.2.1.44), although a candidate cinnamoyl-CoA reductase gene (Tc06_g004070) found in the KEGG database showed high constitutive expression in all samples. Among secondary products, lignin and other compounds associated with the PAL pathway have been most commonly associated with resistance/tolerance to *Phytophthora* in cacao (Ndoumou et al., 1995; Omokolo et al., 2003; Boudjeko et al., 2007; Djocgoue et al., 2007; Louise et al., 2011).

The terpenoid backbone biosynthesis pathway (ko00900), like the PAL-pathway, showed significant induction. Among the secondary products dependent on this pathway are various terpenoids, alkaloids, carotinoids, and N-glycans. The link between JA and ethylene mediated signal transduction and terpene biosynthesis is well established (Dudareva et al., 2006). Terpenoid compound are commonly studied for their activity against herbivores (Mithöfer and Boland, 2012) but the antimicrobial activity of terpenoid compounds have not been studied in any detail in cacao.

## Conclusions

For Ppal, lesion establishment (lesion frequency) and stomatal frequency and pore length were correlated relative to penetration resistance (Iwaro et al., 1997b). Essentially, more frequent and larger pores contribute to susceptibility to Ppal penetration. It is unclear if the same can be said for Pmeg. Our own studies demonstrated that Pmeg zoospores infected directly through appressoria at much higher frequencies than Ppal (Ali et al., 2016), an ability that limits or negates the importance of stomatal frequency during penetration, giving Pmeg an advantage when infecting in the absence of wounds. The added ability of Pmeg to dominate the infection court, even when infecting together, and sporulating first, gives Pmeg another advantage. These basic differences in biology might allow Pmeg to displace Ppal from cacao without the need for Pmeg to have specific advantages over Ppal in dealing with the cacao defense responses during infection.

Overall, the susceptible reactions observed in cacao to the two species are very similar. Only a very few genes, especially where unwounded pod surfaces were used, showed differential expression in a *Phytophthora* species dependent manner. Even these few differences were mostly based on fold change rather than absolute presence or absence of altered expression. Our recent studies of the two *Phytophthora* genomes indicate both species carry large numbers of genes preferentially expressed *in planta* (Ali et al., in press). Of particular interest are the large numbers of genes encoding RXLRs, NPPs, and pectinases showing preferential expression *in planta*. The two species carry similar numbers of *RXLR*-effectors (in fact the largest numbers to date for any *Phytophthora* species) but the Pmeg effector pool may be more diverse. Ppal increased its effector pools, and all its gene families, largely by whole genome duplication while Pmeg appears to have specifically increased gene pools related to pathogenicity including RXLR, CRNs, elicitin, NPPs, and pectinases. As a result, both species carry large numbers of genes putatively encoding proteins capable of suppressing plant defense or causing necrosis in plants either directly or indirectly as a result of enzymatic activity. The ability of RXLRs (Bos et al., 2006; Dou et al., 2008; Wang et al., 2011) and CRNs (Song et al., 2013) to suppress plant defense is well documented as is the ability of elicitins (Yu, 1995) and NPP proteins (Fellbrich et al., 2002; Qutob et al., 2002) to cause necrosis. When considering these elicitors of necrosis along with the many enzymes directly attacking both the plant cell membrane and cell wall, processes known to generate reactive oxygen and stimulate plant defense (O'Brien et al., 2012), there exists more than enough activity to explain the responses seen here which occur predominantly during the necrotrophic phase of the Pmeg/Ppal interaction with susceptible cacao. With this information a firm foundation is formed from which comparisons can be made to reactions in resistant/tolerant cacao genotypes.

## Author contributions

Provided intellectual and editorial comments: BB, SA, MS, and LM. Conceived and designed the experiments: SA and BB. Performed the experiments: SA and MS. Analyzed the data: SA, JS, and DL. Wrote the manuscript: SA and BB.

### Conflict of interest statement

The authors declare that the research was conducted in the absence of any commercial or financial relationships that could be construed as a potential conflict of interest.
