# Supplementary material for: Phytophthora megakarya and P. palmivora, Causal Agents of Black Pod Rot, Induce Similar Plant Defense Responses Late during Infection of Susceptible Cacao Pods
Source: Front Plant Sci. 2017 Feb 14;8:169. doi: 10.3389/fpls.2017.00169 (PMC5306292; doi:10.3389/fpls.2017.00169)
Supplement: Supplementary file 1 [file Table1.DOCX]

**Supplementary Table S1.** Differentially expressed cacao genes of three key metabolic pathways involved in the plant defense in response to Pmeg and Ppal infection.

| **Gene ID*** | **KEGG pathway ID^¥^** | **RNA-Seq RPKM** | | | **Pmeg vs control^±^** | | **Ppal vs control^±^** | |
| --- | --- | --- | --- | --- | --- | --- | --- | --- |
|  |  | **Control** | **Pmeg** | **Ppal** | **Fold change** | **p-value** | **Fold change** | **p-value** |
| ^#^KEGG pathway: **alpha-Linolenic acid metabolism (jasmonate synthesis);** [Induced genes (11)](http://www.kegg.jp/kegg-bin/show_pathway?@ko00592/reference%3dwhite/default%3d%23bfffbf/K14674/K00454/K01723/K10525/K05894/K00232/K10527/K07513/K08241/K18857/K10529) and [Repressed genes (5)](http://www.kegg.jp/kegg-bin/show_pathway?@ko00592/reference%3dwhite/default%3d%23bfffbf/K01723/K05894/K08241/K10528/K18857) | | | | | | | | |
| Tc05_g017570 | [Secretory phospholipase A2 [EC:3.1.1.4]](http://www.kegg.jp/dbget-bin/www_bget?ec:3.1.1.4) | 4902.60 | 12879.85 | 9474.11 | 2.63 | 0.00 | 1.93 | 0.05 |
| Tc04_g000470 | [Alpha-dioxygenase [DOX1]](http://www.kegg.jp/dbget-bin/www_bget?K10529) | 78.86 | 38292.93 | 306467.40 | 485.60 | NA | 3886.40 | NA |
| Tc01_g022950 | [Alcohol dehydrogenase class-P [EC:1.1.1.1]](http://www.kegg.jp/dbget-bin/www_bget?ec:1.1.1.1) | 122.87 | 2220.19 | 28837.06 | 18.07 | NA | 234.70 | NA |
| Tc01_g011540 | [Alcohol dehydrogenase class-P [EC:1.1.1.1]](http://www.kegg.jp/dbget-bin/www_bget?ec:1.1.1.1) | 50.43 | 674.60 | 7645.01 | 13.38 | 0.00 | 151.60 | 0.00 |
| Tc08_g012590 | [Alcohol dehydrogenase class-P [EC:1.1.1.1]](http://www.kegg.jp/dbget-bin/www_bget?ec:1.1.1.1) | 6102.05 | 56906.57 | 173791.87 | 9.33 | 0.00 | 28.48 | 0.00 |
| Tc01_g011550 | [Alcohol dehydrogenase class-P [EC:1.1.1.1]](http://www.kegg.jp/dbget-bin/www_bget?ec:1.1.1.1) | 3319.47 | 10553.89 | 30471.95 | 3.18 | 0.05 | 9.18 | 0.00 |
| Tc08_g012560 | [Alcohol dehydrogenase class-P [EC:1.1.1.1]](http://www.kegg.jp/dbget-bin/www_bget?ec:1.1.1.1) | 90051.38 | 31265.44 | 13712.51 | 0.35 | 0.19 | 0.15 | 0.02 |
| Tc05_g020960 | [Lipoxygenase [EC:1.13.11.12]](http://www.kegg.jp/dbget-bin/www_bget?ec:1.13.11.12) | 5.59 | 113.76 | 591.93 | 20.36 | NA | 105.95 | NA |
| [Tc00_g013400](http://www.kegg.jp/dbget-bin/www_bget?ec:4.2.1.92) | [Lipoxygenase [EC:1.13.11.12]](http://www.kegg.jp/dbget-bin/www_bget?ec:1.13.11.12) | 2190.86 | 66822.47 | 129364.09 | 30.50 | 0.00 | 59.05 | 0.00 |
| Tc08_g001460 | Hydroperoxide dehydratase [EC:4.2.1.92] | 23111.34 | 63897.67 | 86727.83 | 2.76 | 0.02 | 3.75 | 0.00 |
| Tc02_g002090 | Hydroperoxide dehydratase [EC:4.2.1.92] | 1763.41 | 635.44 | 588.85 | 0.36 | 0.01 | 0.33 | 0.00 |
| Tc04_g015220 | [Allene oxide cyclase [EC:5.3.99.6]](http://www.kegg.jp/dbget-bin/www_bget?ec:5.3.99.6) | 3442.59 | 4698.19 | 14317.02 | 1.36 | 0.25 | 4.16 | 0.00 |
| Tc02_g034530 | [Allene oxide cyclase [EC:5.3.99.6]](http://www.kegg.jp/dbget-bin/www_bget?ec:5.3.99.6) | 11402.64 | 23190.03 | 23241.04 | 2.03 | 0.01 | 2.04 | 0.01 |
| Tc03_g009750 | [12-oxophytodienoic acid reductase [EC:1.3.1.42]](http://www.kegg.jp/dbget-bin/www_bget?ec:1.3.1.42) | 0.49 | 126.35 | 348.69 | 258.96 | 0.00 | 714.62 | 0.00 |
| Tc08_g015490 | [12-oxophytodienoic acid reductase [EC:1.3.1.42]](http://www.kegg.jp/dbget-bin/www_bget?ec:1.3.1.42) | 3186.85 | 8929.95 | 6365.81 | 2.80 | 0.00 | 2.00 | 0.03 |
| Tc02_g001340 | [12-oxophytodienoic acid reductase [EC:1.3.1.42]](http://www.kegg.jp/dbget-bin/www_bget?ec:1.3.1.42) | 86.39 | 25.32 | 12.06 | 0.29 | 0.02 | 0.14 | 0.00 |
| Tc05_g002270 | Acyl-CoA oxidase [ACX] | 21308.19 | 38430.48 | 60324.00 | 1.80 | 0.16 | 2.83 | 0.01 |
| Tc09_g020600 | Acyl-CoA oxidase [ACX] | 14530.97 | 31611.37 | 34729.95 | 2.18 | 0.02 | 2.39 | 0.01 |
| Tc01_g009270 | Acyl-CoA oxidase [ACX] | 10392.18 | 55036.06 | 71432.73 | 5.30 | 0.00 | 6.87 | 0.00 |
| Tc09_g003740 | [Enoyl-CoA hydratase/3-hydroxyacyl-CoA dehydrogenase [MFP2]](http://www.kegg.jp/dbget-bin/www_bget?K10527) | 10953.72 | 337185.93 | 494011.54 | 30.78 | 0.00 | 45.10 | 0.00 |
| Tc01_g022760 | [acetyl-CoA acyltransferase [EC:2.3.1.16]](http://www.kegg.jp/dbget-bin/www_bget?ec:2.3.1.16) | 1391.53 | 2735.46 | 3114.09 | 1.97 | 0.00 | 2.24 | 0.00 |
| Tc08_g002470 | Jasmonate O-methyltransferase [EC:2.1.1.141] | 0.00 | 11.93 | 61.86 | 11.93 | 0.55 | 61.86 | 0.11 |
| Tc10_g001820 | Jasmonate O-methyltransferase [EC:2.1.1.141] | 1849.54 | 823.63 | 794.42 | 0.45 | 0.02 | 0.43 | 0.02 |
| Tc08_g002490 | Jasmonate O-methyltransferase [EC:2.1.1.141] | 12.02 | 2.34 | 0.77 | 0.19 | 0.05 | 0.06 | 0.00 |
| ^#^KEGG pathway: **Cysteine and methionine metabolism (ethylene synthesis);** [Induced genes (13)](http://www.kegg.jp/kegg-bin/show_pathway?@ko00270/reference%3dwhite/default%3d%23bfffbf/K00640/K00547/K00789/K01611/K00899/K01762/K05933/K00928/K00826/K14454/K14455/K00016/K01761) and [Repressed genes (7)](http://www.kegg.jp/kegg-bin/show_pathway?@ko00270/reference%3dwhite/default%3d%23bfffbf/K01738/K08967/K00815/K00558/K00928/K01739/K00026) | | | | | | | | |
| Tc01_g032660 | [S-adenosylmethionine synthetase [EC:2.5.1.6]](http://www.kegg.jp/dbget-bin/www_bget?ec:2.5.1.6) | 42543.20 | 102018.52 | 123236.50 | 2.40 | 0.00 | 2.90 | 0.00 |
| Tc04_g030460 | [S-adenosylmethionine synthetase [EC:2.5.1.6]](http://www.kegg.jp/dbget-bin/www_bget?ec:2.5.1.6) | 10804.23 | 20672.01 | 22959.12 | 1.91 | 0.08 | 2.13 | 0.04 |
| Tc01_g007030 | [1-aminocyclopropane-1-carboxylate synthase [EC:4.4.1.14]](http://www.kegg.jp/dbget-bin/www_bget?ec:4.4.1.14) | 38.24 | 273.54 | 209.40 | 7.15 | 0.00 | 5.48 | 0.00 |
| Tc01_g035360 | [1-aminocyclopropane-1-carboxylate synthase [EC:4.4.1.14]](http://www.kegg.jp/dbget-bin/www_bget?ec:4.4.1.14) | 25.02 | 11023.24 | 11698.10 | 440.49 | 0.00 | 467.46 | 0.00 |
| Tc09_g008260 | [1-aminocyclopropane-1-carboxylate synthase [EC:4.4.1.14]](http://www.kegg.jp/dbget-bin/www_bget?ec:4.4.1.14) | 1.95 | 68.07 | 93.17 | 34.87 | 0.00 | 47.74 | 0.00 |
| Tc08_g006930 | [1-aminocyclopropane-1-carboxylate synthase [EC:4.4.1.14]](http://www.kegg.jp/dbget-bin/www_bget?ec:4.4.1.14) | 0.00 | 21.12 | 2.89 | 21.12 | NA | 2.89 | NA |
| Tc03_g027980 | [1-aminocyclopropane-1-carboxylate synthase [EC:4.4.1.14]](http://www.kegg.jp/dbget-bin/www_bget?ec:4.4.1.14) | 4078.86 | 9476.09 | 16851.40 | 2.32 | 0.00 | 4.13 | 0.00 |
| Tc02_g030910 | [S-adenosylmethionine decarboxylase [EC:4.1.1.50]](http://www.kegg.jp/dbget-bin/www_bget?ec:4.1.1.50) | 8283.01 | 25408.25 | 28727.15 | 3.07 | 0.00 | 3.47 | 0.00 |
| Tc01_g017710 | [Aminocyclopropanecarboxylate oxidase [EC:1.14.17.4]](http://www.kegg.jp/dbget-bin/www_bget?ec:1.14.17.4) | 110684.05 | 387663.95 | 435491.55 | 3.50 | 0.00 | 3.93 | 0.00 |
| Tc04_g015650 | [S-adenosylmethionine decarboxylase [EC:4.1.1.50]](http://www.kegg.jp/dbget-bin/www_bget?ec:4.1.1.50) | 8193.53 | 16045.41 | 17684.09 | 1.96 | 0.03 | 2.16 | 0.01 |
| Tc09_g008060 | [Aminocyclopropanecarboxylate oxidase [EC:1.14.17.4]](http://www.kegg.jp/dbget-bin/www_bget?ec:1.14.17.4) | 202.30 | 824.97 | 6977.11 | 4.08 | NA | 34.49 | NA |
| ^#^KEGG pathway: **Plant-pathogen interaction;** [Induced genes (16)](http://www.kegg.jp/kegg-bin/show_pathway?@ko04626/reference%3dwhite/default%3d%23bfffbf/K13412/K13447/K05391/K02183/K13448/K13420/K13414/K13413/K13424/K13425/K13449/K13456/K13457/K04079/K13459/K13429) and [Repressed genes (5)](http://www.kegg.jp/kegg-bin/show_pathway?@ko04626/reference%3dwhite/default%3d%23bfffbf/K02183/K13448/K13427/K13420/K04368) | | | | | | | | |
| Tc02_g002400 | [Pathogenesis-related protein 1 [PR1]](http://www.kegg.jp/dbget-bin/www_bget?K13449) | 0.00 | 1.44 | 4.26 | 1.44 | 0.17 | 4.26 | 0.02 |
| Tc02_g002390 | [Pathogenesis-related protein 1 [PR1]](http://www.kegg.jp/dbget-bin/www_bget?K13449) | 1.12 | 116.08 | 398.64 | 103.29 | NA | 354.71 | NA |
| Tc02_g002420 | [Pathogenesis-related protein 1 [PR1]](http://www.kegg.jp/dbget-bin/www_bget?K13449) | 0.00 | 53.27 | 127.45 | 53.27 | 0.00 | 127.45 | 0.00 |
| Tc02_g002410 | [Pathogenesis-related protein 1 [PR1]](http://www.kegg.jp/dbget-bin/www_bget?K13449) | 16890.01 | 249256.25 | 353914.10 | 14.76 | 0.00 | 20.95 | 0.00 |
| Tc01_g035330 | [WRKY transcription factor 29 [WRKY29]](http://www.kegg.jp/dbget-bin/www_bget?K13426+K13425) | 939.37 | 6049.06 | 10976.29 | 6.44 | 0.00 | 11.68 | 0.00 |
| Tc06_g002590 | [Mitogen-activated protein kinase kinase 4/5 [MKK4/5]](http://www.kegg.jp/dbget-bin/www_bget?ec:2.7.12.2) | 5912.45 | 14989.70 | 13902.12 | 2.54 | 0.01 | 2.35 | 0.01 |
| Tc09_g008710 | [Mitogen-activated protein kinase kinase 1 [MKK1/2]](http://www.kegg.jp/dbget-bin/www_bget?ec:2.7.12.2) | 113.12 | 8.44 | 6.46 | 0.07 | 0.00 | 0.06 | 0.00 |
| Tc08_g009940 | [Mitogen-activated protein kinase kinase kinase 1 [MEKK1]](http://www.kegg.jp/dbget-bin/www_bget?ec:2.7.11.25) | 5262.22 | 16328.10 | 13431.75 | 3.10 | 0.00 | 2.55 | 0.02 |
| Tc07_g010600 | [LRR receptor-like serine/threonine-protein kinase [FLS2]](http://www.kegg.jp/dbget-bin/www_bget?ec:2.7.11.1) | 6441.65 | 9058.93 | 17155.49 | 1.41 | 0.42 | 2.66 | 0.02 |
| Tc06_g013970 | [LRR receptor-like serine/threonine-protein kinase [FLS2]](http://www.kegg.jp/dbget-bin/www_bget?ec:2.7.11.1) | 418.95 | 2248.17 | 3626.75 | 5.37 | 0.00 | 8.66 | 0.00 |
| Tc00_g081080 | [LRR receptor-like serine/threonine-protein kinase [FLS2]](http://www.kegg.jp/dbget-bin/www_bget?ec:2.7.11.1) | 240.22 | 126.10 | 95.38 | 0.52 | 0.00 | 0.40 | 0.00 |
| Tc09_g034740 | [WRKY transcription factor 25 [WRKY25]](http://www.kegg.jp/dbget-bin/www_bget?K13423+K13424) | 31081.29 | 110173.50 | 121174.93 | 3.54 | 0.00 | 3.90 | 0.00 |
| Tc02_g033160 | [Calmodulin [CALM]](http://www.kegg.jp/dbget-bin/www_bget?K02183+K13448) | 71.42 | 365.21 | 286.74 | 5.11 | 0.00 | 4.01 | 0.00 |
| Tc00_g092430 | [Calmodulin [CALM]](http://www.kegg.jp/dbget-bin/www_bget?K02183+K13448) | 423.89 | 18683.14 | 25421.80 | 44.08 | 0.00 | 59.97 | 0.00 |
| Tc04_g029700 | [Calmodulin [CALM]](http://www.kegg.jp/dbget-bin/www_bget?K02183+K13448) | 2054.25 | 32273.55 | 16977.18 | 15.71 | 0.00 | 8.26 | 0.00 |
| Tc04_g009190 | [Calmodulin [CALM]](http://www.kegg.jp/dbget-bin/www_bget?K02183+K13448) | 1308.21 | 9255.63 | 9985.69 | 7.08 | 0.00 | 7.63 | 0.00 |
| Tc01_g034560 | [Cyclic nucleotide gated channel [CNGF]](http://www.kegg.jp/dbget-bin/www_bget?K05391) | 3204.30 | 22712.65 | 37264.49 | 7.09 | 0.00 | 11.63 | 0.00 |
| Tc03_g028110 | [Respiratory burst oxidase [RBOH]](http://www.kegg.jp/dbget-bin/www_bget?K13447) | 3691.23 | 8483.16 | 9392.12 | 2.30 | 0.04 | 2.54 | 0.02 |
| Tc05_g030130 | [Respiratory burst oxidase [RBOH]](http://www.kegg.jp/dbget-bin/www_bget?K13447) | 97.90 | 8413.89 | 46801.25 | 85.94 | 0.00 | 478.05 | 0.00 |
| Tc09_g006400 | [Respiratory burst oxidase [RBOH]](http://www.kegg.jp/dbget-bin/www_bget?K13447) | 0.58 | 8.68 | 36.89 | 15.07 | 0.00 | 64.07 | 0.00 |
| Tc08_g002020 | [Respiratory burst oxidase [RBOH]](http://www.kegg.jp/dbget-bin/www_bget?K13447) | 6070.41 | 59343.34 | 121658.81 | 9.78 | 0.00 | 20.04 | 0.00 |
| Tc08_g004170 | Calcium-dependent protein kinase [CDPK] | 139.70 | 189.84 | 632.33 | 1.36 | 0.60 | 4.53 | 0.01 |
| Tc02_g012480 | Calcium-dependent protein kinase [CDPK] | 8407.86 | 37957.23 | 36904.04 | 4.51 | 0.00 | 4.39 | 0.00 |
| Tc00_g010560 | Calcium-dependent protein kinase [CDPK] | 3625.98 | 9386.98 | 10124.96 | 2.59 | 0.01 | 2.79 | 0.01 |
| Tc07_g003300 | Calcium-dependent protein kinase [CDPK] | 7453.92 | 19882.08 | 16088.39 | 2.67 | 0.00 | 2.16 | 0.01 |
| Tc00_g034230 | Calcium-dependent protein kinase [CDPK] | 114.03 | 12066.21 | 43996.63 | 105.82 | NA | 385.84 | NA |
| Tc04_g018630 | Calcium-dependent protein kinase [CDPK] | 0.00 | 14.44 | 54.43 | 14.44 | 0.00 | 54.43 | 0.00 |
| Tc00_g081380 | Calcium-dependent protein kinase [CDPK] | 5573.90 | 31254.29 | 50102.44 | 5.61 | 0.00 | 8.99 | 0.00 |
| Tc03_g028230 | Calcium-dependent protein kinase [CDPK] | 1675.62 | 4275.11 | 3168.49 | 2.55 | 0.01 | 1.89 | 0.06 |
| Tc03_g017170 | [Nitric-oxide synthase, plant [NOS]](http://www.kegg.jp/dbget-bin/www_bget?ec:1.14.13.39) | 515.86 | 231.35 | 229.28 | 0.45 | 0.02 | 0.44 | 0.02 |
| Tc09_g008710 | [Mitogen-activated protein kinase kinase 1 [MKK1/2]](http://www.kegg.jp/dbget-bin/www_bget?ec:2.7.12.2) | 113.12 | 8.44 | 6.46 | 0.07 | 0.00 | 0.06 | 0.00 |
| Tc03_g019880 | [Heat shock protein [HSP90]](http://www.kegg.jp/dbget-bin/www_bget?K09487+K04079) | 1482.24 | 6010.76 | 8311.00 | 4.06 | 0.00 | 5.61 | 0.00 |
| Tc00_g047990 | [RPM1-interacting protein 4 [RIN4]](http://www.kegg.jp/dbget-bin/www_bget?K13456) | 6459.87 | 23299.28 | 26730.34 | 3.61 | 0.00 | 4.14 | 0.00 |
| Tc04_g011730 | [Disease resistance protein [RPM1]](http://www.kegg.jp/dbget-bin/www_bget?K13457) | 704.87 | 2269.17 | 3735.72 | 3.22 | 0.00 | 5.30 | 0.00 |
| Tc03_g030250 | [Disease resistance protein [RPM1]](http://www.kegg.jp/dbget-bin/www_bget?K13457) | 3.80 | 582.38 | 713.12 | 153.28 | 0.00 | 187.69 | 0.00 |
| Tc03_g030240 | [Disease resistance protein [RPM1]](http://www.kegg.jp/dbget-bin/www_bget?K13457) | 1698.36 | 27077.54 | 26474.30 | 15.94 | 0.00 | 15.59 | 0.00 |
| Tc05_g022610 | [Disease resistance protein [RPM1]](http://www.kegg.jp/dbget-bin/www_bget?K13457) | 397.14 | 1340.01 | 871.66 | 3.37 | 0.01 | 2.19 | 0.09 |
| Tc09_g007540 | [Disease resistance protein [RPS2]](http://www.kegg.jp/dbget-bin/www_bget?K13459) | 829.29 | 2166.11 | 2168.31 | 2.61 | 0.01 | 2.61 | 0.01 |
| Tc07_g003420 | [Disease resistance protein [RPS2]](http://www.kegg.jp/dbget-bin/www_bget?K13459) | 2.16 | 15.23 | 4.34 | 7.05 | 0.00 | 2.01 | 0.34 |
| Tc01_g016180 | [Chitin elicitor receptor kinase 1 [CERK1]](http://www.kegg.jp/dbget-bin/www_bget?K13429) | 4245.30 | 14770.90 | 16459.36 | 3.48 | 0.00 | 3.88 | 0.00 |
| Tc02_g001780 | [Serine/threonine-protein kinase [PBS1]](http://www.kegg.jp/dbget-bin/www_bget?K13430) | 343.35 | 1988.13 | 2391.22 | 5.79 | 0.00 | 6.96 | 0.00 |
| Tc00_g073450 | [Serine/threonine-protein kinase [PBS1]](http://www.kegg.jp/dbget-bin/www_bget?K13430) | 177.34 | 1232.72 | 1196.25 | 6.95 | 0.00 | 6.75 | 0.00 |
| Tc10_g010540 | [Disease resistance protein [RPS2]](http://www.kegg.jp/dbget-bin/www_bget?K13459) | 260.87 | 513.89 | 826.71 | 1.97 | 0.03 | 3.17 | 0.00 |
| Tc00_g012290 | [Serine/threonine-protein kinase [PBS1]](http://www.kegg.jp/dbget-bin/www_bget?K13430) | 44.02 | 137.37 | 266.98 | 3.12 | 0.01 | 6.06 | 0.00 |
| Tc02_g001870 | [Serine/threonine-protein kinase [PBS1]](http://www.kegg.jp/dbget-bin/www_bget?K13430) | 41.61 | 1681.12 | 2563.92 | 40.41 | 0.00 | 61.62 | 0.00 |
| ^#^KEGG pathway: **Plant hormone signal transduction;** [Induced genes (22)](http://www.kegg.jp/kegg-bin/show_pathway?@ko04075/reference%3dwhite/default%3d%23bfffbf/K14484/K14487/K14488/K14490/K14491/K14492/K14495/K14496/K14497/K14498/K14432/K14509/K13413/K14515/K14516/K14504/K13463/K13464/K13422/K14508/K14431/K13449) and [Repressed genes (14)](http://www.kegg.jp/kegg-bin/show_pathway?@ko04075/reference%3dwhite/default%3d%23bfffbf/K13946/K14484/K14487/K14488/K14489/K14490/K14492/K16189/K14497/K14432/K13415/K14499/K14504/K14505) | | | | | | | | |
| Tc06_g011480 | [Regulatory protein [NPR1]](http://www.kegg.jp/dbget-bin/www_bget?K14508) | 6285.95 | 14838.41 | 12346.70 | 2.36 | 0.00 | 1.96 | 0.00 |
| Tc00_g028220 | [Transcription factor [TGA]](http://www.kegg.jp/dbget-bin/www_bget?K14431) | 159.76 | 535.45 | 2044.27 | 3.35 | NA | 12.80 | NA |
| Tc02_g002400 | [Pathogenesis-related protein 1 [Pr-1]](http://www.kegg.jp/dbget-bin/www_bget?K13449) | 0.00 | 1.44 | 4.26 | 1.44 | 0.17 | 4.26 | 0.02 |
| Tc02_g002390 | [Pathogenesis-related protein 1 [Pr-1]](http://www.kegg.jp/dbget-bin/www_bget?K13449) | 1.12 | 116.08 | 398.64 | 103.29 | NA | 354.71 | NA |
| Tc02_g002420 | [Pathogenesis-related protein 1 [Pr-1]](http://www.kegg.jp/dbget-bin/www_bget?K13449) | 0.00 | 53.27 | 127.45 | 53.27 | 0.00 | 127.45 | 0.00 |
| Tc02_g002410 | [Pathogenesis-related protein 1 [Pr-1]](http://www.kegg.jp/dbget-bin/www_bget?K13449) | 16890.01 | 249256.25 | 353914.10 | 14.76 | 0.00 | 20.95 | 0.00 |
| Tc06_g014010 | [Coronatine-insensitive protein 1 [COI1]](http://www.kegg.jp/dbget-bin/www_bget?K13463) | 0.00 | 5.48 | 1.53 | 5.48 | 0.03 | 1.53 | 0.24 |
| Tc09_g006620 | [Jasmonate ZIM domain-containing protein [JAZ]](http://www.kegg.jp/dbget-bin/www_bget?K13464) | 4134.32 | 16065.82 | 20409.05 | 3.89 | 0.00 | 4.94 | 0.00 |
| Tc02_g019970 | [Jasmonate ZIM domain-containing protein [JAZ]](http://www.kegg.jp/dbget-bin/www_bget?K13464) | 464.55 | 3194.53 | 8510.36 | 6.88 | 0.00 | 18.32 | 0.00 |
| Tc06_g010210 | [Jasmonate ZIM domain-containing protein [JAZ]](http://www.kegg.jp/dbget-bin/www_bget?K13464) | 1506.36 | 11576.81 | 16558.54 | 7.69 | 0.00 | 10.99 | 0.00 |
| Tc03_g023110 | [Transcription factor MYC2](http://www.kegg.jp/dbget-bin/www_bget?K13422) | 9203.56 | 20910.27 | 23369.22 | 2.27 | 0.03 | 2.54 | 0.01 |
| Tc04_g025010 | [Ethylene receptor [ETR]](http://www.kegg.jp/dbget-bin/www_bget?K14509) | 18731.49 | 37611.15 | 44236.95 | 2.01 | 0.07 | 2.36 | 0.03 |
| Tc06_g002590 | [Mitogen-activated protein kinase kinase 4/5 [SIMKK]](http://www.kegg.jp/dbget-bin/www_bget?K13413) | 5912.45 | 14989.70 | 13902.12 | 2.54 | 0.01 | 2.35 | 0.01 |
| Tc09_g011440 | [EIN3-binding F-box protein [EBF1/2]](http://www.kegg.jp/dbget-bin/www_bget?K14515) | 16041.65 | 20609.90 | 35556.49 | 1.28 | 0.45 | 2.22 | 0.02 |
| Tc00_g030730 | [Ethylene-responsive transcription factor 1 [ERF1/2]](http://www.kegg.jp/dbget-bin/www_bget?K14516+K14517) | 697.52 | 1499.76 | 2647.15 | 2.15 | 0.00 | 3.80 | 0.00 |
| Tc04_g025410 | [Ethylene-responsive transcription factor 1 [ERF1/2]](http://www.kegg.jp/dbget-bin/www_bget?K14516+K14517) | 209.95 | 3310.58 | 4184.97 | 15.77 | 0.00 | 19.93 | 0.00 |
| Tc05_g026470 | [Ethylene-responsive transcription factor 1 [ERF1/2]](http://www.kegg.jp/dbget-bin/www_bget?K14516+K14517) | 17.31 | 118.45 | 116.77 | 6.84 | 0.00 | 6.74 | 0.00 |
| Tc05_g026480 | [Ethylene-responsive transcription factor 1 [ERF1/2]](http://www.kegg.jp/dbget-bin/www_bget?K14516+K14517) | 0.00 | 1.44 | 12.87 | 1.44 | 0.11 | 12.87 | 0.00 |
| Tc03_g023150 | [Abscisic acid receptor PYR/PYL family](http://www.kegg.jp/dbget-bin/www_bget?K14496) | 1100.28 | 1990.16 | 3166.44 | 1.81 | 0.13 | 2.88 | 0.01 |
| Tc05_g001720 | [Abscisic acid receptor PYR/PYL family](http://www.kegg.jp/dbget-bin/www_bget?K14496) | 967.96 | 2060.41 | 2762.60 | 2.13 | 0.10 | 2.85 | 0.02 |
| Tc10_g000870 | [Abscisic acid receptor PYR/PYL family](http://www.kegg.jp/dbget-bin/www_bget?K14496) | 909.75 | 2949.37 | 2687.53 | 3.24 | 0.01 | 2.95 | 0.02 |
| Tc09_g004740 | [Abscisic acid receptor PYR/PYL family](http://www.kegg.jp/dbget-bin/www_bget?K14496) | 0.00 | 0.58 | 6.54 | 0.58 | 0.61 | 6.54 | 0.04 |
| Tc03_g018960 | [Protein phosphatase 2C [PP2C]](http://www.kegg.jp/dbget-bin/www_bget?ec:3.1.3.16) | 1519.33 | 1570.38 | 4320.17 | 1.03 | 0.90 | 2.84 | 0.00 |
| Tc03_g017400 | [Protein phosphatase 2C [PP2C]](http://www.kegg.jp/dbget-bin/www_bget?ec:3.1.3.16) | 3215.28 | 1288.44 | 2468.00 | 0.40 | 0.00 | 0.77 | 0.40 |
| Tc03_g024070 | [serine/threonine-protein kinase SRK2](http://www.kegg.jp/dbget-bin/www_bget?ec:2.7.11.1) | 1535.90 | 4566.13 | 7018.92 | 2.97 | 0.00 | 4.57 | 0.00 |
| Tc00_g054140 | [ABA responsive element binding factor [ABF]](http://www.kegg.jp/dbget-bin/www_bget?K14432) | 2.62 | 11.76 | 13.07 | 4.50 | 0.06 | 5.00 | 0.04 |
| Tc05_g008870 | [ABA responsive element binding factor [ABF]](http://www.kegg.jp/dbget-bin/www_bget?K14432) | 145.64 | 69.43 | 118.93 | 0.48 | 0.00 | 0.82 | 0.42 |
| Tc02_g029810 | [Arabidopsis histidine kinase 2/3/4 (cytokinin receptor) [CRE1]](http://www.kegg.jp/dbget-bin/www_bget?ec:2.7.13.3) | 748.49 | 356.93 | 512.78 | 0.48 | 0.01 | 0.69 | 0.20 |
| Tc05_g010310 | [Histidine-containing phosphotransfer peotein [APH]](http://www.kegg.jp/dbget-bin/www_bget?K14490) | 28.28 | 35.51 | 119.85 | 1.26 | 0.66 | 4.24 | 0.00 |
| Tc09_g004300 | [Histidine-containing phosphotransfer peotein [APH]](http://www.kegg.jp/dbget-bin/www_bget?K14490) | 139.63 | 390.56 | 280.69 | 2.80 | 0.01 | 2.01 | 0.09 |
| Tc05_g028800 | [Histidine-containing phosphotransfer peotein [APH]](http://www.kegg.jp/dbget-bin/www_bget?K14490) | 132.83 | 19.57 | 19.42 | 0.15 | 0.01 | 0.15 | 0.01 |
| Tc04_g009060 | [Histidine-containing phosphotransfer peotein [APH]](http://www.kegg.jp/dbget-bin/www_bget?K14490) | 9.09 | 0.58 | 2.89 | 0.06 | 0.01 | 0.32 | 0.16 |
| Tc04_g027120 | [Histidine-containing phosphotransfer peotein [APH]](http://www.kegg.jp/dbget-bin/www_bget?K14490) | 195.14 | 65.23 | 33.82 | 0.33 | 0.01 | 0.17 | 0.00 |
| Tc09_g003070 | [Two-component response regulator ARR-B family](http://www.kegg.jp/dbget-bin/www_bget?K14491) | 109.06 | 181.02 | 330.76 | 1.66 | 0.19 | 3.03 | 0.00 |
| Tc03_g000310 | [Two-component response regulator ARR-A family](http://www.kegg.jp/dbget-bin/www_bget?K14492) | 180.66 | 479.97 | 653.58 | 2.66 | 0.08 | 3.62 | 0.02 |
| Tc10_g014960 | [Two-component response regulator ARR-A family](http://www.kegg.jp/dbget-bin/www_bget?K14492) | 277.46 | 885.85 | 1721.41 | 3.19 | 0.01 | 6.20 | 0.00 |
| Tc08_g011180 | [Two-component response regulator ARR-A family](http://www.kegg.jp/dbget-bin/www_bget?K14492) | 1045.96 | 483.24 | 190.02 | 0.46 | 0.27 | 0.18 | 0.02 |
| Tc08_g010200 | [Auxin influx carrier (AUX1 LAX family)](http://www.kegg.jp/dbget-bin/www_bget?K13946) | 30.89 | 7.54 | 11.33 | 0.24 | 0.00 | 0.37 | 0.03 |
| Tc10_g001900 | [Auxin influx carrier (AUX1 LAX family)](http://www.kegg.jp/dbget-bin/www_bget?K13946) | 739.22 | 233.07 | 123.50 | 0.32 | 0.02 | 0.17 | 0.00 |
| Tc07_g008010 | [Auxin responsive GH3](http://www.kegg.jp/dbget-bin/www_bget?K14487) | 48.80 | 11.92 | 8.30 | 0.24 | 0.05 | 0.17 | 0.01 |
| Tc07_g008050 | [Auxin responsive GH3](http://www.kegg.jp/dbget-bin/www_bget?K14487) | 682.40 | 203.25 | 100.81 | 0.30 | 0.04 | 0.15 | 0.00 |
| Tc02_g018750 | [Auxin responsive GH3](http://www.kegg.jp/dbget-bin/www_bget?K14487) | 8392.84 | 17229.56 | 26557.35 | 2.05 | 0.14 | 3.16 | 0.02 |
| Tc07_g008110 | [Auxin responsive GH3](http://www.kegg.jp/dbget-bin/www_bget?K14487) | 0.00 | 21.78 | 211.38 | 21.78 | 0.00 | 211.38 | 0.00 |
| Tc01_g005970 | [Auxin responsive GH3](http://www.kegg.jp/dbget-bin/www_bget?K14487) | 243.78 | 4032.33 | 5385.89 | 16.54 | 0.00 | 22.09 | 0.00 |
| Tc02_g009780 | [Auxin responsive GH3](http://www.kegg.jp/dbget-bin/www_bget?K14487) | 82.20 | 776.45 | 1456.89 | 9.45 | 0.00 | 17.72 | 0.00 |
| Tc04_g024590 | [Auxin-responsive protein [AUX/IAA]](http://www.kegg.jp/dbget-bin/www_bget?K14484) | 70.14 | 265.21 | 1321.07 | 3.78 | 0.01 | 18.83 | 0.00 |
| Tc09_g011790 | [Auxin-responsive protein [AUX/IAA]](http://www.kegg.jp/dbget-bin/www_bget?K14484) | 269.24 | 133.55 | 44.75 | 0.50 | 0.17 | 0.17 | 0.00 |
| Tc05_g027350 | [Auxin-responsive protein [AUX/IAA]](http://www.kegg.jp/dbget-bin/www_bget?K14484) | 2553.15 | 1587.16 | 796.55 | 0.62 | 0.42 | 0.31 | 0.05 |
| Tc04_g026430 | [Auxin-responsive protein [AUX/IAA]](http://www.kegg.jp/dbget-bin/www_bget?K14484) | 78.45 | 70.42 | 24.24 | 0.90 | 0.81 | 0.31 | 0.01 |
| Tc00_g023090 | [Auxin-responsive protein [AUX/IAA]](http://www.kegg.jp/dbget-bin/www_bget?K14484) | 291.64 | 185.55 | 74.99 | 0.64 | 0.37 | 0.26 | 0.01 |
| Tc09_g009400 | [Auxin-responsive protein [AUX/IAA]](http://www.kegg.jp/dbget-bin/www_bget?K14484) | 847.50 | 435.23 | 184.12 | 0.51 | 0.32 | 0.22 | 0.03 |
| Tc01_g032980 | [Auxin-responsive protein [AUX/IAA]](http://www.kegg.jp/dbget-bin/www_bget?K14484) | 19.45 | 21.42 | 59.08 | 1.10 | 0.83 | 3.04 | 0.01 |
| Tc09_g006380 | [SAUR family protein](file:///C:\Users\AliS\AppData\Local\Microsoft\AppData\Local\Microsoft\Windows\Temporary%20Internet%20Files\Content.Outlook\TZPM7XT9\SAUR%20family%20protein) | 32.00 | 75.74 | 111.62 | 2.37 | 0.06 | 3.49 | 0.01 |
| Tc00_g079470 | [SAUR family protein](file:///C:\Users\AliS\AppData\Local\Microsoft\AppData\Local\Microsoft\Windows\Temporary%20Internet%20Files\Content.Outlook\TZPM7XT9\SAUR%20family%20protein) | 3405.48 | 5573.75 | 8666.57 | 1.64 | 0.20 | 2.54 | 0.02 |
| Tc03_g025540 | [SAUR family protein](file:///C:\Users\AliS\AppData\Local\Microsoft\AppData\Local\Microsoft\Windows\Temporary%20Internet%20Files\Content.Outlook\TZPM7XT9\SAUR%20family%20protein) | 116.87 | 421.20 | 464.79 | 3.60 | 0.00 | 3.98 | 0.00 |
| Tc02_g005950 | [SAUR family protein](file:///C:\Users\AliS\AppData\Local\Microsoft\AppData\Local\Microsoft\Windows\Temporary%20Internet%20Files\Content.Outlook\TZPM7XT9\SAUR%20family%20protein) | 19.28 | 91.94 | 59.56 | 4.77 | 0.00 | 3.09 | 0.00 |
| Tc02_g005820 | [SAUR family protein](file:///C:\Users\AliS\AppData\Local\Microsoft\AppData\Local\Microsoft\Windows\Temporary%20Internet%20Files\Content.Outlook\TZPM7XT9\SAUR%20family%20protein) | 109.43 | 372.98 | 1025.38 | 3.41 | 0.00 | 9.37 | 0.00 |
| Tc03_g021540 | [SAUR family protein](file:///C:\Users\AliS\AppData\Local\Microsoft\AppData\Local\Microsoft\Windows\Temporary%20Internet%20Files\Content.Outlook\TZPM7XT9\SAUR%20family%20protein) | 2939.64 | 10104.93 | 17912.05 | 3.44 | 0.01 | 6.09 | 0.00 |
| Tc04_g019610 | [SAUR family protein](file:///C:\Users\AliS\AppData\Local\Microsoft\AppData\Local\Microsoft\Windows\Temporary%20Internet%20Files\Content.Outlook\TZPM7XT9\SAUR%20family%20protein) | 0.00 | 10.34 | 8.84 | 10.34 | 0.00 | 8.84 | 0.00 |
| Tc02_g005970 | [SAUR family protein](file:///C:\Users\AliS\AppData\Local\Microsoft\AppData\Local\Microsoft\Windows\Temporary%20Internet%20Files\Content.Outlook\TZPM7XT9\SAUR%20family%20protein) | 2.19 | 19.78 | 14.41 | 9.03 | 0.01 | 6.57 | 0.03 |
| Tc05_g009340 | [SAUR family protein](file:///C:\Users\AliS\AppData\Local\Microsoft\AppData\Local\Microsoft\Windows\Temporary%20Internet%20Files\Content.Outlook\TZPM7XT9\SAUR%20family%20protein) | 10.03 | 112.51 | 31.52 | 11.22 | 0.00 | 3.14 | 0.13 |
| Tc03_g015370 | [SAUR family protein](file:///C:\Users\AliS\AppData\Local\Microsoft\AppData\Local\Microsoft\Windows\Temporary%20Internet%20Files\Content.Outlook\TZPM7XT9\SAUR%20family%20protein) | 55.39 | 126.35 | 981.01 | 2.28 | 0.13 | 17.71 | 0.00 |
| Tc02_g005850 | [SAUR family protein](file:///C:\Users\AliS\AppData\Local\Microsoft\AppData\Local\Microsoft\Windows\Temporary%20Internet%20Files\Content.Outlook\TZPM7XT9\SAUR%20family%20protein) | 1.12 | 7.97 | 17.54 | 7.09 | 0.06 | 15.61 | 0.00 |
| Tc02_g005880 | [SAUR family protein](file:///C:\Users\AliS\AppData\Local\Microsoft\AppData\Local\Microsoft\Windows\Temporary%20Internet%20Files\Content.Outlook\TZPM7XT9\SAUR%20family%20protein) | 0.55 | 3.99 | 7.58 | 7.29 | 0.28 | 13.83 | 0.11 |
| Tc02_g005990 | [SAUR family protein](file:///C:\Users\AliS\AppData\Local\Microsoft\AppData\Local\Microsoft\Windows\Temporary%20Internet%20Files\Content.Outlook\TZPM7XT9\SAUR%20family%20protein) | 0.00 | 2.03 | 5.40 | 2.03 | 0.11 | 5.40 | 0.01 |
| Tc02_g006210 | [SAUR family protein](file:///C:\Users\AliS\AppData\Local\Microsoft\AppData\Local\Microsoft\Windows\Temporary%20Internet%20Files\Content.Outlook\TZPM7XT9\SAUR%20family%20protein) | 402.87 | 147.92 | 120.80 | 0.37 | 0.02 | 0.30 | 0.01 |
| Tc06_g011150 | [SAUR family protein](file:///C:\Users\AliS\AppData\Local\Microsoft\AppData\Local\Microsoft\Windows\Temporary%20Internet%20Files\Content.Outlook\TZPM7XT9\SAUR%20family%20protein) | 17.45 | 4.92 | 10.34 | 0.28 | 0.02 | 0.59 | 0.28 |
| Tc01_g033970 | [SAUR family protein](file:///C:\Users\AliS\AppData\Local\Microsoft\AppData\Local\Microsoft\Windows\Temporary%20Internet%20Files\Content.Outlook\TZPM7XT9\SAUR%20family%20protein) | 118.71 | 29.79 | 53.16 | 0.25 | 0.04 | 0.45 | 0.22 |
| Tc02_g006130 | [SAUR family protein](file:///C:\Users\AliS\AppData\Local\Microsoft\AppData\Local\Microsoft\Windows\Temporary%20Internet%20Files\Content.Outlook\TZPM7XT9\SAUR%20family%20protein) | 9.13 | 4.67 | 1.45 | 0.51 | 0.40 | 0.16 | 0.04 |
| Tc01_g010390 | [Brassinosteroid insensitive 1 [BRI1]](http://www.kegg.jp/dbget-bin/www_bget?K13415) | 11305.75 | 5198.12 | 4912.33 | 0.46 | 0.03 | 0.43 | 0.02 |
| Tc08_g000700 | [BRI1 kinase inhibitor 1 [BKI1]](http://www.kegg.jp/dbget-bin/www_bget?K14499) | 1819.16 | 838.93 | 825.82 | 0.46 | 0.01 | 0.45 | 0.01 |
| Tc04_g029260 | [xyloglucan:xyloglucosyl transferase [TCH4]](http://www.kegg.jp/dbget-bin/www_bget?ec:2.4.1.207) | 18.38 | 91.60 | 180.59 | 4.98 | 0.00 | 9.83 | 0.00 |
| Tc09_g016940 | [xyloglucan:xyloglucosyl transferase [TCH4]](http://www.kegg.jp/dbget-bin/www_bget?ec:2.4.1.207) | 87.31 | 4107.27 | 1677.40 | 47.04 | NA | 19.21 | NA |
| Tc09_g016910 | [xyloglucan:xyloglucosyl transferase [TCH4]](http://www.kegg.jp/dbget-bin/www_bget?ec:2.4.1.207) | 59.53 | 630.07 | 364.60 | 10.58 | 0.00 | 6.12 | 0.00 |
| Tc02_g009300 | [Cyclin D3 [CYCD3]](http://www.kegg.jp/dbget-bin/www_bget?K14505) | 182.69 | 21.02 | 7.03 | 0.12 | 0.00 | 0.04 | 0.00 |
| Tc08_g008070 | [F-box protein GID2](http://www.kegg.jp/dbget-bin/www_bget?K14495) | 1915.93 | 3912.09 | 3693.52 | 2.04 | 0.01 | 1.93 | 0.02 |
| **^*^**Cacao gene Ids are based on *T*. *cacao* genome (Argout *et al.*, 2010) http://cocoagendb.cirad.fr/gbrowse/cgi-bin/gbrowse/theobroma/  #Click once to follow the pathway. Enzymes marked in green are the differentially expressed based on the homology with transcribed amino acid sequence of the cacao gene.  ^¥^ Click once to follow the gene/enzyme description.  ^±^Fold change highlighted as green indicates induction and blue indicates repression in response to Pmeg/Ppal infection. *P*-value highlighted as yellow are ≥ 0.05. | | | | | | | | |
