# Supplementary material for: Phytophthora megakarya and P. palmivora, Causal Agents of Black Pod Rot, Induce Similar Plant Defense Responses Late during Infection of Susceptible Cacao Pods
Source: Front Plant Sci. 2017 Feb 14;8:169. doi: 10.3389/fpls.2017.00169 (PMC5306292; doi:10.3389/fpls.2017.00169)
Supplement: Supplementary file 2 [file Table2.DOCX]

**Supplementary Table S2.** Differentially expressed cacao genes of endocytosis pathway in response to Pmeg and Ppal infection.

| **Gene ID*** | **KEGG pathway ID^¥^** | **RNA-Seq RPKM** | | | **Pmeg vs control^±^** | | **Ppal vs control^±^** | |
| --- | --- | --- | --- | --- | --- | --- | --- | --- |
|  |  | **Control** | **Pmeg** | **Ppal** | **Fold change** | **p-value** | **Fold change** | **p-value** |
| ^#^KEGG pathway: **Endocytosis**; [Induced genes (17)](http://www.kegg.jp/kegg-bin/show_pathway?@ko04144/reference%3dwhite/default%3d%23bfffbf/K01528/K00889/K01115/K11824/K12471/K07897/K12183/K12185/K12191/K19476/K12200/K07889/K07904/K12486/K12492/K18442/K07937) and [Repressed genes (3)](http://www.kegg.jp/kegg-bin/show_pathway?@ko04144/reference%3dwhite/default%3d%23bfffbf/K12191/K19476/K07904) | | | | | | | | |
| Tc06_g000130 | [Dynamin GTPase](http://www.kegg.jp/dbget-bin/www_bget?K01528) | 4387.927 | 9517.65 | 9760.326 | 2.17 | 0.03 | 2.22 | 0.02 |
| Tc09_g031790 | [Epsin](http://www.kegg.jp/dbget-bin/www_bget?K12471) | 130.1276 | 219.4112 | 558.3159 | 1.69 | 0.18 | 4.29 | 0.00 |
| Tc09_g029040 | [AP-2 complex subunit alpha](http://www.kegg.jp/dbget-bin/www_bget?K11824+K11825+K11826+K11827) | 5178.122 | 10181.59 | 10854.86 | 1.97 | 0.05 | 2.10 | 0.03 |
| Tc10_g014710 | [1-phosphatidylinositol-4-phosphate 5-kinase [PIP5K]](http://www.kegg.jp/dbget-bin/www_bget?K00889+K13712) | 9.039087 | 12.41458 | 23.43943 | 1.37 | 0.46 | 2.59 | 0.02 |
| Tc08_g004280 | [Phospholipase D1/2 [PLD]](http://www.kegg.jp/dbget-bin/www_bget?K01115) | 1088.143 | 1835.309 | 2671.303 | 1.69 | 0.16 | 2.45 | 0.02 |
| Tc01_g037000 | [Phospholipase D1/2 [PLD]](http://www.kegg.jp/dbget-bin/www_bget?K01115) | 8105.857 | 17491.04 | 16574.91 | 2.16 | 0.02 | 2.04 | 0.03 |
| Tc03_g026510 | [Phospholipase D1/2 [PLD]](http://www.kegg.jp/dbget-bin/www_bget?K01115) | 340.6184 | 14267.4 | 25728.55 | 41.89 | 0.00 | 75.53 | 0.00 |
| Tc09_g011400 | [Phospholipase D1/2 [PLD]](http://www.kegg.jp/dbget-bin/www_bget?K01115) | 7.966641 | 633.9723 | 128.7844 | 79.58 | 0.00 | 16.17 | 0.00 |
| Tc06_g009470 | [Phospholipase D1/2 [PLD]](http://www.kegg.jp/dbget-bin/www_bget?K01115) | 13.11841 | 236.082 | 19.08835 | 18.00 | 0.00 | 1.46 | 0.39 |
| Tc01_g014080 | [Actin related protein 2/3 complex [Arp2/3]](http://www.kegg.jp/dbget-bin/www_bget?K05757+K05758+K05756+K05755+K05754) | 1219.878 | 2384.274 | 1461.131 | 1.95 | 0.00 | 1.20 | 0.36 |
| Tc02_g000020 | [Actin related protein 2/3 complex [Arp2/3]](http://www.kegg.jp/dbget-bin/www_bget?K05757+K05758+K05756+K05755+K05754) | 3799.487 | 5313.419 | 5156.293 | 1.40 | 0.03 | 1.36 | 0.05 |
| Tc04_g001110 | [ADP-ribosylation factor 1 [ARF]](http://www.kegg.jp/dbget-bin/www_bget?K07937+K07938+K07940+K07941) | 11872.64 | 25853.99 | 28958.62 | 2.18 | 0.01 | 2.44 | 0.00 |
| Tc06_g016720 | [Stromal membrane-associated protein [ArfGAP]](http://www.kegg.jp/dbget-bin/www_bget?K12486+K05737+K12487+K12488+K12489+K18439+K18440+K12490+K12491+K17848+K12492+K12493) | 2818.396 | 7088.043 | 7997.456 | 2.51 | 0.00 | 2.84 | 0.00 |
| Tc02_g012630 | [Brefeldin A-inhibited GEF [ArfGEF]](http://www.kegg.jp/dbget-bin/www_bget?ko:K18442) | 5297.791 | 12078.72 | 12627.9 | 2.28 | 0.02 | 2.38 | 0.01 |
| Tc04_g029120 | [STAM-binding protein [AMSH]](http://www.kegg.jp/dbget-bin/www_bget?K11866) | 2006.883 | 3318.475 | 3251.682 | 1.65 | 0.04 | 1.62 | 0.05 |
| Tc01_g010230 | [Ras-related protein Rab-7A](http://www.kegg.jp/dbget-bin/www_bget?K07897) | 108.8584 | 76.32774 | 364.0046 | 0.70 | 0.34 | 3.34 | 0.00 |
| Tc09_g009710 | [Ras-related protein Rab-7A](http://www.kegg.jp/dbget-bin/www_bget?K07897) | 812.4768 | 1433.673 | 1426.729 | 1.76 | 0.01 | 1.76 | 0.01 |
| Tc06_g008500 | [Ras-related protein Rab-7A](http://www.kegg.jp/dbget-bin/www_bget?K07897) | 3740.476 | 4784.376 | 5474.908 | 1.28 | 0.14 | 1.46 | 0.02 |
| Tc05_g009640 | [Sorting nexin-1/2 [SNX1/2]](http://www.kegg.jp/dbget-bin/www_bget?K17917) | 3651.13 | 5898.039 | 5788.777 | 1.62 | 0.02 | 1.59 | 0.02 |
| Tc00_g023240 | [ESCRT-I complex subunit TSG101](http://www.kegg.jp/dbget-bin/www_bget?K12183) | 384.4732 | 2057.623 | 3956.683 | 5.35 | 0.00 | 10.29 | 0.00 |
| Tc10_g013050 | [ESCRT-I complex subunit TSG101](http://www.kegg.jp/dbget-bin/www_bget?K12183) | 5032.734 | 11813.99 | 9646.399 | 2.35 | 0.01 | 1.92 | 0.03 |
| Tc05_g007580 | [ESCRT-I complex subunit VPS37](http://www.kegg.jp/dbget-bin/www_bget?K12185) | 3967.049 | 8058.263 | 7340.317 | 2.03 | 0.01 | 1.85 | 0.02 |
| Tc06_g016750 | [ESCRT-I complex subunit VPS28](http://www.kegg.jp/dbget-bin/www_bget?K12184) | 3633.155 | 7179.356 | 6699.386 | 1.98 | 0.01 | 1.84 | 0.02 |
| Tc00_g035750 | [Programmed cell death 6-interacting protein [Alix]](http://www.kegg.jp/dbget-bin/www_bget?K12200) | 11310.27 | 25485.01 | 20586.88 | 2.25 | 0.01 | 1.82 | 0.06 |
| Tc09_g009920 | [Ras-related protein Rab-5A](http://www.kegg.jp/dbget-bin/www_bget?K07887+K07888+K07889) | 2699.426 | 5487.121 | 6923.531 | 2.03 | 0.01 | 2.56 | 0.00 |
| Tc03_g030090 | [ESCRT-II complex subunit VPS25](http://www.kegg.jp/dbget-bin/www_bget?K12189) | 2792.458 | 4378.593 | 4611.569 | 1.57 | 0.02 | 1.65 | 0.01 |
| Tc07_g002600 | [EH domain-containing protein 1 [EHD1]](http://www.kegg.jp/dbget-bin/www_bget?K12483) | 2330.915 | 3554.906 | 3235.151 | 1.53 | 0.00 | 1.39 | 0.01 |
| Tc09_g030970 | [Ras-related protein Rab-11A](http://www.kegg.jp/dbget-bin/www_bget?K07904+K07905) | 2131.885 | 3657.819 | 4485.071 | 1.72 | 0.03 | 2.10 | 0.00 |
| Tc10_g002370 | [Ras-related protein Rab-11A](http://www.kegg.jp/dbget-bin/www_bget?K07904+K07905) | 0 | 4.606975 | 11.91763 | 4.61 | 0.03 | 11.92 | 0.00 |
| Tc05_g018180 | [Ras-related protein Rab-11A](http://www.kegg.jp/dbget-bin/www_bget?K07904+K07905) | 56.30489 | 139.5379 | 394.9481 | 2.48 | 0.02 | 7.01 | 0.00 |
| Tc06_g015050 | [Ras-related protein Rab-11A](http://www.kegg.jp/dbget-bin/www_bget?K07904+K07905) | 1356.235 | 5526.089 | 9019.129 | 4.07 | 0.00 | 6.65 | 0.00 |
| Tc04_g019970 | [Ras-related protein Rab-11A](http://www.kegg.jp/dbget-bin/www_bget?K07904+K07905) | 130.6422 | 54.59497 | 61.78784 | 0.42 | 0.00 | 0.47 | 0.01 |
| Tc01_g034070 | [Ras-related protein Rab-11A](http://www.kegg.jp/dbget-bin/www_bget?K07904+K07905) | 96.83944 | 37.35952 | 25.57802 | 0.39 | 0.09 | 0.26 | 0.02 |
| Tc04_g014920 | [Vacuolar protein sorting-associated protein IST1](http://www.kegg.jp/dbget-bin/www_bget?K19476) | 875.3998 | 3118.483 | 4335.881 | 3.56 | 0.00 | 4.95 | 0.00 |
| Tc09_g008290 | [Vacuolar protein sorting-associated protein IST1](http://www.kegg.jp/dbget-bin/www_bget?K19476) | 5930.162 | 16111.34 | 15322.79 | 2.72 | 0.01 | 2.58 | 0.01 |
| Tc02_g034280 | [Vacuolar protein sorting-associated protein IST1](http://www.kegg.jp/dbget-bin/www_bget?K19476) | 10768.88 | 22262.96 | 23250.66 | 2.07 | 0.04 | 2.16 | 0.03 |
| Tc06_g016240 | [Vacuolar protein sorting-associated protein IST1](http://www.kegg.jp/dbget-bin/www_bget?K19476) | 0.487937 | 22.75322 | 5.320409 | 46.63 | 0.00 | 10.90 | 0.01 |
| Tc00_g050280 | [Vacuolar protein sorting-associated protein IST1](http://www.kegg.jp/dbget-bin/www_bget?K19476) | 3697.024 | 7469.372 | 6604.17 | 2.02 | 0.01 | 1.79 | 0.04 |
| Tc01_g000900 | [Vacuolar protein sorting-associated protein IST1](http://www.kegg.jp/dbget-bin/www_bget?K19476) | 126.2026 | 21.42111 | 5.801438 | 0.17 | 0.00 | 0.05 | 0.00 |
| Tc02_g015820 | [Vacuolar protein sorting-associated protein IST1](http://www.kegg.jp/dbget-bin/www_bget?K19476) | 889.1625 | 246.1971 | 344.1686 | 0.28 | 0.00 | 0.39 | 0.01 |
| Tc06_g009180 | [Charged multivesicular body protein 1 [CHMP1]](http://www.kegg.jp/dbget-bin/www_bget?K12197) | 16001.3 | 27683.22 | 26330.61 | 1.73 | 0.03 | 1.65 | 0.04 |
| Tc09_g007260 | [Charged multivesicular body protein 2A [CHMP2]](http://www.kegg.jp/dbget-bin/www_bget?K12191+K12192) | 8289.158 | 21833.77 | 22665 | 2.63 | 0.00 | 2.73 | 0.00 |
| Tc06_g000520 | [Charged multivesicular body protein 2A [CHMP2]](http://www.kegg.jp/dbget-bin/www_bget?K12191+K12192) | 118.0193 | 14.50689 | 5.024325 | 0.12 | 0.00 | 0.04 | 0.00 |
| Tc09_g028030 | [Vacuolar protein-sorting-associated protein 4 [VPS4]](http://www.kegg.jp/dbget-bin/www_bget?K12196) | 13506.83 | 24466.41 | 23449.3 | 1.81 | 0.02 | 1.74 | 0.03 |
| **^*^**Cacao gene Ids are based on *T*. *cacao* genome (Argout *et al.*, 2010) http://cocoagendb.cirad.fr/gbrowse/cgi-bin/gbrowse/theobroma/  #Click once to follow the pathway. Enzymes marked in green are the differentially expressed based on the homology with transcribed amino acid sequence of the cacao gene.  ^¥^ Click once to follow the gene/enzyme description.  ^±^Fold change highlighted as green indicates induction and blue indicates repression in response to Pmeg/Ppal infection. P-value highlighted as yellow are ≥ 0.05. | | | | | | | | |
