# Supplementary material for: Phytophthora megakarya and P. palmivora, Causal Agents of Black Pod Rot, Induce Similar Plant Defense Responses Late during Infection of Susceptible Cacao Pods
Source: Front Plant Sci. 2017 Feb 14;8:169. doi: 10.3389/fpls.2017.00169 (PMC5306292; doi:10.3389/fpls.2017.00169)
Supplement: Supplementary file 3 [file Table3.DOCX]

**Supplementary Table S3.** Differentially expressed cacao genes of three key metabolic pathways involved in secondary metabolites synthesis in response to Pmeg and Ppal infection.

| **Gene ID*** | **KEGG pathway ID^¥^** | **RNA-Seq RPKM** | | | **Pmeg vs control^±^** | | **Ppal vs control^±^** | | |
| --- | --- | --- | --- | --- | --- | --- | --- | --- | --- |
|  |  | **Control** | **Pmeg** | **Ppal** | **Fold change** | **p-value** | **Fold change** | **p-value** | |
| **^#^**KEGG pathway: **Phenylpropanoid biosynthesis**; [Induced genes (13)](http://www.kegg.jp/kegg-bin/show_pathway?@ko00940/reference%3dwhite/default%3d%23bfffbf/K10775/K01188/K05349/K05350/K01904/K00487/K00083/K00430/K13065/K09754/K18368/K13066/K00588) and [Repressed genes (7)](http://www.kegg.jp/kegg-bin/show_pathway?@ko00940/reference%3dwhite/default%3d%23bfffbf/K01188/K05350/K01904/K00083/K00430/K13065/K13066) | | | | | | | | | |
| Tc10_g005730 | [Phenylalanine ammonia-lyase [EC:4.3.1.24]](http://www.kegg.jp/dbget-bin/www_bget?ec:4.3.1.24) | 76555.86 | 147783.9 | 277313.6 | 1.93 | 0.14 | 3.62 | 0.00 | |
| Tc00_g004780 | [Phenylalanine ammonia-lyase [EC:4.3.1.24]](http://www.kegg.jp/dbget-bin/www_bget?ec:4.3.1.24) | 3793.634 | 6339.222 | 10327.64 | 1.67 | 0.12 | 2.72 | 0.00 | |
| Tc05_g006550 | [Phenylalanine ammonia-lyase [EC:4.3.1.24]](http://www.kegg.jp/dbget-bin/www_bget?ec:4.3.1.24) | 38475.53 | 246084.5 | 403224.5 | 6.40 | 0.00 | 10.48 | 0.00 | |
| Tc03_g030380 | [4-coumarate--CoA ligase [EC:6.2.1.12]](http://www.kegg.jp/dbget-bin/www_bget?ec:6.2.1.12) | 119.2283 | 359.2429 | 890.0934 | 3.01 | 0.01 | 7.47 | 0.00 | |
| Tc06_g010940 | [4-coumarate--CoA ligase [EC:6.2.1.12]](http://www.kegg.jp/dbget-bin/www_bget?ec:6.2.1.12) | 10442.59 | 97039.37 | 190376.5 | 9.29 | 0.00 | 18.23 | 0.00 | |
| Tc00_g081720 | [4-coumarate--CoA ligase [EC:6.2.1.12]](http://www.kegg.jp/dbget-bin/www_bget?ec:6.2.1.12) | 3786.504 | 1492.974 | 1745.911 | 0.39 | 0.00 | 0.46 | 0.00 | |
| Tc09_g035230 | [Trans-cinnamate 4-monooxygenase [EC:1.14.13.11]](http://www.kegg.jp/dbget-bin/www_bget?ec:1.14.13.11) | 34040.52 | 137445.4 | 234781.9 | 4.04 | 0.00 | 6.90 | 0.00 | |
| Tc09_g021750 | [Trans-cinnamate 4-monooxygenase [EC:1.14.13.11]](http://www.kegg.jp/dbget-bin/www_bget?ec:1.14.13.11) | 3040.194 | 44559.89 | 98324.3 | 14.66 | 0.00 | 32.34 | 0.00 | |
| Tc09_g002490 | [Shikimate O-hydroxycinnamoyltransferase [EC:2.3.1.133]](http://www.kegg.jp/dbget-bin/www_bget?ec:2.3.1.133) | 28.21643 | 88.64492 | 123.8366 | 3.14 | 0.01 | 4.39 | 0.00 | |
| Tc06_g004820 | [Shikimate O-hydroxycinnamoyltransferase [EC:2.3.1.133]](http://www.kegg.jp/dbget-bin/www_bget?ec:2.3.1.133) | 25537.32 | 82613.6 | 108473.3 | 3.24 | 0.00 | 4.25 | 0.00 | |
| Tc00_g076740 | [Shikimate O-hydroxycinnamoyltransferase [EC:2.3.1.133]](http://www.kegg.jp/dbget-bin/www_bget?ec:2.3.1.133) | 296.5245 | 3144.266 | 18099.92 | 10.60 | NA | 61.04 | NA | |
| Tc05_g006310 | [Shikimate O-hydroxycinnamoyltransferase [EC:2.3.1.133]](http://www.kegg.jp/dbget-bin/www_bget?ec:2.3.1.133) | 9.326357 | 9.56102 | 2.169842 | 1.03 | 0.95 | 0.23 | 0.03 | |
| Tc05_g013970 | [Coumaroylquinate(coumaroylshikimate) 3'-monooxygenase [EC:1.14.13.36]](http://www.kegg.jp/dbget-bin/www_bget?ec:1.14.13.36) | 27029.59 | 113172.1 | 157974.2 | 4.19 | 0.00 | 5.84 | 0.00 | |
| Tc06_g006820 | [Caffeoylshikimate esterase [EC:3.1.1.-]](http://www.kegg.jp/dbget-bin/www_bget?ko:K18368) | 20521.02 | 57692.61 | 81365.97 | 2.81 | 0.00 | 3.97 | 0.00 | |
| Tc05_g023880 | [Caffeic acid 3-O-methyltransferase [EC:2.1.1.68]](http://www.kegg.jp/dbget-bin/www_bget?ec:2.1.1.68) | 33.455 | 76.40066 | 95.40703 | 2.28 | 0.07 | 2.85 | 0.02 | |
| Tc05_g023860 | [Caffeic acid 3-O-methyltransferase [EC:2.1.1.68]](http://www.kegg.jp/dbget-bin/www_bget?ec:2.1.1.68) | 4845.446 | 8007.63 | 11406.04 | 1.65 | 0.15 | 2.35 | 0.01 | |
| Tc03_g022520 | [Caffeic acid 3-O-methyltransferase [EC:2.1.1.68]](http://www.kegg.jp/dbget-bin/www_bget?ec:2.1.1.68) | 11735.75 | 32660.61 | 47091.57 | 2.78 | 0.00 | 4.01 | 0.00 | |
| Tc08_g011220 | [Caffeic acid 3-O-methyltransferase [EC:2.1.1.68]](http://www.kegg.jp/dbget-bin/www_bget?ec:2.1.1.68) | 1637.851 | 830.8424 | 742.3037 | 0.51 | 0.01 | 0.45 | 0.00 | |
| Tc00_g018080 | [Caffeic acid 3-O-methyltransferase [EC:2.1.1.68]](http://www.kegg.jp/dbget-bin/www_bget?ec:2.1.1.68) | 236.0385 | 105.7824 | 91.08875 | 0.45 | 0.06 | 0.39 | 0.02 | |
| Tc01_g005290 | [Caffeoyl-CoA O-methyltransferase [EC:2.1.1.104]](http://www.kegg.jp/dbget-bin/www_bget?ec:2.1.1.104) | 1007.387 | 2630.668 | 3672.486 | 2.61 | 0.03 | 3.65 | 0.00 | |
| Tc01_g007730 | [Cinnamyl-alcohol dehydrogenase [EC:1.1.1.195]](http://www.kegg.jp/dbget-bin/www_bget?ec:1.1.1.195) | 3230.25 | 8001.406 | 11084.83 | 2.48 | 0.04 | 3.43 | 0.01 | |
| Tc08_g004030 | [Cinnamyl-alcohol dehydrogenase [EC:1.1.1.195]](http://www.kegg.jp/dbget-bin/www_bget?ec:1.1.1.195) | 345.6861 | 1107.572 | 900.9341 | 3.20 | 0.00 | 2.61 | 0.01 | |
| Tc01_g005740 | [Cinnamyl-alcohol dehydrogenase [EC:1.1.1.195]](http://www.kegg.jp/dbget-bin/www_bget?ec:1.1.1.195) | 3.947368 | 512.134 | 456.0192 | 129.74 | 0.00 | 115.52 | 0.00 | |
| Tc01_g005730 | [Cinnamyl-alcohol dehydrogenase [EC:1.1.1.195]](http://www.kegg.jp/dbget-bin/www_bget?ec:1.1.1.195) | 0 | 53.1131 | 31.27743 | 53.11 | 0.00 | 31.28 | 0.00 | |
| Tc02_g009500 | [Cinnamyl-alcohol dehydrogenase [EC:1.1.1.195]](http://www.kegg.jp/dbget-bin/www_bget?ec:1.1.1.195) | 132985 | 69245.28 | 65711.03 | 0.52 | 0.01 | 0.49 | 0.00 | |
| Tc00_g045940 | [Peroxidase [EC:1.11.1.7]](http://www.kegg.jp/dbget-bin/www_bget?ec:1.11.1.7) | 830.1102 | 2139.344 | 4102.586 | 2.58 | 0.10 | 4.94 | 0.00 | |
| Tc04_g029820 | [Peroxidase [EC:1.11.1.7]](http://www.kegg.jp/dbget-bin/www_bget?ec:1.11.1.7) | 14.2304 | 31.19773 | 44.09831 | 2.19 | 0.12 | 3.10 | 0.03 | |
| Tc09_g017500 | [Peroxidase [EC:1.11.1.7]](http://www.kegg.jp/dbget-bin/www_bget?ec:1.11.1.7) | 21.77781 | 71.21975 | 59.72795 | 3.27 | 0.00 | 2.74 | 0.01 | |
| Tc06_g008540 | [Peroxidase [EC:1.11.1.7]](http://www.kegg.jp/dbget-bin/www_bget?ec:1.11.1.7) | 53.74094 | 184.5493 | 125.2136 | 3.43 | 0.00 | 2.33 | 0.00 | |
| Tc04_g016340 | [Peroxidase [EC:1.11.1.7]](http://www.kegg.jp/dbget-bin/www_bget?ec:1.11.1.7) | 5.15479 | 452.5699 | 22555.8 | 87.80 | NA | 4375.70 | NA | |
| Tc04_g016740 | [Peroxidase [EC:1.11.1.7]](http://www.kegg.jp/dbget-bin/www_bget?ec:1.11.1.7) | 72.50148 | 6998.936 | 110325 | 96.54 | NA | 1521.69 | NA | |
| Tc02_g029110 | [Peroxidase [EC:1.11.1.7]](http://www.kegg.jp/dbget-bin/www_bget?ec:1.11.1.7) | 6.866988 | 645.5643 | 7263.996 | 94.01 | 0.02 | 1057.81 | 0.00 | |
| Tc02_g029120 | [Peroxidase [EC:1.11.1.7]](http://www.kegg.jp/dbget-bin/www_bget?ec:1.11.1.7) | 4.947429 | 538.1786 | 4751.536 | 108.78 | 0.00 | 960.41 | 0.00 | |
| Tc01_g032570 | [Peroxidase [EC:1.11.1.7]](http://www.kegg.jp/dbget-bin/www_bget?ec:1.11.1.7) | 6.611254 | 246.1712 | 3455.2 | 37.24 | 0.00 | 522.62 | 0.00 | |
| Tc04_g016760 | [Peroxidase [EC:1.11.1.7]](http://www.kegg.jp/dbget-bin/www_bget?ec:1.11.1.7) | 222.7942 | 6243.982 | 82257.99 | 28.03 | NA | 369.21 | NA | |
| Tc02_g029090 | [Peroxidase [EC:1.11.1.7]](http://www.kegg.jp/dbget-bin/www_bget?ec:1.11.1.7) | 221.2299 | 6666.853 | 62981.53 | 30.14 | 0.00 | 284.69 | 0.00 | |
| Tc00_g045400 | [Peroxidase [EC:1.11.1.7]](http://www.kegg.jp/dbget-bin/www_bget?ec:1.11.1.7) | 2192.511 | 109606.5 | 55010.83 | 49.99 | 0.00 | 25.09 | 0.00 | |
| Tc00_g054480 | [Peroxidase [EC:1.11.1.7]](http://www.kegg.jp/dbget-bin/www_bget?ec:1.11.1.7) | 0.57585 | 5.781367 | 12.58904 | 10.04 | 0.02 | 21.86 | 0.00 | |
| Tc09_g034910 | [Peroxidase [EC:1.11.1.7]](http://www.kegg.jp/dbget-bin/www_bget?ec:1.11.1.7) | 214.2352 | 2111.046 | 2470.077 | 9.85 | 0.00 | 11.53 | 0.00 | |
| Tc02_g012020 | [Peroxidase [EC:1.11.1.7]](http://www.kegg.jp/dbget-bin/www_bget?ec:1.11.1.7) | 2835.383 | 7734.877 | 5800.283 | 2.73 | 0.01 | 2.05 | 0.08 | |
| Tc09_g011360 | [Peroxidase [EC:1.11.1.7]](http://www.kegg.jp/dbget-bin/www_bget?ec:1.11.1.7) | 21.30925 | 70.81521 | 22.13799 | 3.32 | 0.01 | 1.04 | 0.93 | |
| Tc00_g045440 | [Peroxidase [EC:1.11.1.7]](http://www.kegg.jp/dbget-bin/www_bget?ec:1.11.1.7) | 11.95355 | 50.18012 | 3.881445 | 4.20 | 0.04 | 0.32 | 0.18 | |
| Tc09_g000620 | [Peroxidase [EC:1.11.1.7]](http://www.kegg.jp/dbget-bin/www_bget?ec:1.11.1.7) | 4.287341 | 27.16889 | 7.843967 | 6.34 | 0.00 | 1.83 | 0.33 | |
| Tc04_g028060 | [Peroxidase [EC:1.11.1.7]](http://www.kegg.jp/dbget-bin/www_bget?ec:1.11.1.7) | 1.72755 | 4.838277 | 2775.915 | 2.80 | NA | 1606.85 | NA | |
| Tc00_g045630 | [Peroxidase [EC:1.11.1.7]](http://www.kegg.jp/dbget-bin/www_bget?ec:1.11.1.7) | 1103.672 | 3759.067 | 44142.1 | 3.41 | NA | 40.00 | NA | |
| Tc00_g020190 | [Peroxidase [EC:1.11.1.7]](http://www.kegg.jp/dbget-bin/www_bget?ec:1.11.1.7) | 5.147441 | 4.887984 | 76.25215 | 0.95 | 0.95 | 14.81 | 0.00 | |
| Tc06_g019150 | [Peroxidase [EC:1.11.1.7]](http://www.kegg.jp/dbget-bin/www_bget?ec:1.11.1.7) | 297.73 | 49.93034 | 22.96252 | 0.17 | 0.01 | 0.08 | 0.00 | |
| Tc10_g016080 | [Peroxidase [EC:1.11.1.7]](http://www.kegg.jp/dbget-bin/www_bget?ec:1.11.1.7) | 30.87564 | 1.439047 | 0.684641 | 0.05 | 0.00 | 0.02 | 0.00 | |
| Tc01_g013330 | [Peroxidase [EC:1.11.1.7]](http://www.kegg.jp/dbget-bin/www_bget?ec:1.11.1.7) | 1011.439 | 410.7337 | 305.3835 | 0.41 | 0.03 | 0.30 | 0.00 | |
| Tc00_g045610 | [Peroxidase [EC:1.11.1.7]](http://www.kegg.jp/dbget-bin/www_bget?ec:1.11.1.7) | 8621.08 | 3537.815 | 2153.121 | 0.41 | 0.04 | 0.25 | 0.00 | |
| Tc03_g021880 | [Peroxidase [EC:1.11.1.7]](http://www.kegg.jp/dbget-bin/www_bget?ec:1.11.1.7) | 634.8086 | 228.9192 | 156.7861 | 0.36 | 0.00 | 0.25 | 0.00 | |
| Tc09_g034950 | [Peroxidase [EC:1.11.1.7]](http://www.kegg.jp/dbget-bin/www_bget?ec:1.11.1.7) | 154.8913 | 73.06328 | 78.97898 | 0.47 | 0.01 | 0.51 | 0.02 | |
| Tc01_g006280 | [Peroxidase [EC:1.11.1.7]](http://www.kegg.jp/dbget-bin/www_bget?ec:1.11.1.7) | 56254.15 | 26733.25 | 41836.47 | 0.48 | 0.02 | 0.74 | 0.34 | |
| Tc03_g000530 | [Peroxidase [EC:1.11.1.7]](http://www.kegg.jp/dbget-bin/www_bget?ec:1.11.1.7) | 117.7152 | 27.13577 | 53.68434 | 0.23 | 0.01 | 0.46 | 0.17 | |
| Tc04_g000340 | [Peroxidase [EC:1.11.1.7]](http://www.kegg.jp/dbget-bin/www_bget?ec:1.11.1.7) | 820.9826 | 429.4897 | 332.1754 | 0.52 | 0.12 | 0.40 | 0.03 | |
| Tc10_g016040 | [Peroxidase [EC:1.11.1.7]](http://www.kegg.jp/dbget-bin/www_bget?ec:1.11.1.7) | 88.4627 | 31.39591 | 20.84435 | 0.35 | 0.08 | 0.24 | 0.02 | |
| Tc10_g015970 | [Peroxidase [EC:1.11.1.7]](http://www.kegg.jp/dbget-bin/www_bget?ec:1.11.1.7) | 15357.03 | 6143.47 | 3535.117 | 0.40 | 0.05 | 0.23 | 0.00 | |
| Tc09_g022210 | [beta-glucosidase [EC:3.2.1.21]](http://www.kegg.jp/dbget-bin/www_bget?ec:3.2.1.21) | 128.8972 | 137.2388 | 319.5635 | 1.06 | 0.85 | 2.48 | 0.01 | |
| Tc09_g022250 | [beta-glucosidase [EC:3.2.1.21]](http://www.kegg.jp/dbget-bin/www_bget?ec:3.2.1.21) | 52.20938 | 6446.512 | 21292.12 | 123.47 | NA | 407.82 | NA | |
| Tc01_g030460 | [beta-glucosidase [EC:3.2.1.21]](http://www.kegg.jp/dbget-bin/www_bget?ec:3.2.1.21) | 3219.914 | 1546.63 | 1512.999 | 0.48 | 0.03 | 0.47 | 0.02 | |
| Tc01_g030490 | [beta-glucosidase [EC:3.2.1.21]](http://www.kegg.jp/dbget-bin/www_bget?ec:3.2.1.21) | 2558.924 | 1120.435 | 1079.736 | 0.44 | 0.03 | 0.42 | 0.03 | |
| Tc05_g019680 | [beta-glucosidase [EC:3.2.1.21]](http://www.kegg.jp/dbget-bin/www_bget?ec:3.2.1.21) | 243.2516 | 61.12212 | 60.74817 | 0.25 | 0.01 | 0.25 | 0.01 | |
| **^#^**KEGG pathway: **Phenylalanine, tyrosine and tryptophan biosynthesis**; [Induced genes (12)](http://www.kegg.jp/kegg-bin/show_pathway?@ko00400/reference%3dwhite/default%3d%23bfffbf/K01626/K01735/K13832/K00891/K00800/K01736/K01696/K15227/K05359/K14454/K14455/K15849) and [Repressed genes (3)](http://www.kegg.jp/kegg-bin/show_pathway?@ko00400/reference%3dwhite/default%3d%23bfffbf/K01657/K05359/K00815) | | | | | | | | | |
| Tc08_g008780 | [3-deoxy-7-phosphoheptulonate synthase [EC:2.5.1.54]](http://www.kegg.jp/dbget-bin/www_bget?ec:2.5.1.54) | 29504.88 | 115495 | 168095.8 | 3.91 | 0.00 | 5.70 | | 0.00 |
| Tc01_g001360 | [3-dehydroquinate synthase [EC:4.2.3.4]](http://www.kegg.jp/dbget-bin/www_bget?ec:4.2.3.4) | 14199.43 | 28523.77 | 32741.3 | 2.01 | 0.01 | 2.31 | | 0.00 |
| Tc05_g024370 | [3-dehydroquinate dehydratase I [EC:4.2.1.10]](http://www.kegg.jp/dbget-bin/www_bget?ec:4.2.1.10) | 95.45335 | 219.4147 | 270.1008 | 2.30 | 0.04 | 2.83 | | 0.01 |
| Tc05_g024370 | [Shikimate dehydrogenase [EC:1.1.1.25]](http://www.kegg.jp/dbget-bin/www_bget?ec:1.1.1.25) | 95.45335 | 219.4147 | 270.1008 | 2.30 | 0.04 | 2.83 | | 0.01 |
| Tc01_g010070 | [Shikimate kinase [EC:2.7.1.71]](http://www.kegg.jp/dbget-bin/www_bget?ec:2.7.1.71) | 3421.69 | 7400.879 | 10237.12 | 2.16 | 0.02 | 2.99 | | 0.00 |
| Tc01_g037810 | [3-phosphoshikimate 1-carboxyvinyltransferase [EC:2.5.1.19]](http://www.kegg.jp/dbget-bin/www_bget?ec:2.5.1.19) | 10716.31 | 31650.82 | 36222.67 | 2.95 | 0.00 | 3.38 | | 0.00 |
| Tc10_g005370 | [Chorismate synthase [EC:4.2.3.5]](http://www.kegg.jp/dbget-bin/www_bget?ec:4.2.3.5) | 11307.73 | 28514.18 | 31392.15 | 2.52 | 0.00 | 2.78 | | 0.00 |
| Tc09_g028840 | [Prephenate dehydratase [EC:4.2.1.51]](http://www.kegg.jp/dbget-bin/www_bget?ec:4.2.1.51) | 6495.158 | 51607.52 | 71683.33 | 7.95 | 0.00 | 11.04 | | 0.00 |
| Tc02_g034990 | [Prephenate dehydratase [EC:4.2.1.51]](http://www.kegg.jp/dbget-bin/www_bget?ec:4.2.1.51) | 1234.41 | 591.5389 | 764.4452 | 0.48 | 0.00 | 0.62 | | 0.06 |
| Tc01_g009420 | [Bifunctional aspartate aminotransferase and glutamate/aspartate-prephenate aminotransferase [EC:2.6.1.1/78/79]](http://www.kegg.jp/dbget-bin/www_bget?ko:K15849) | 13072.31 | 29403.93 | 32721.21 | 2.25 | 0.01 | 2.50 | | 0.00 |
| Tc09_g009580 | [Aspartate aminotransferase, cytoplasmic [EC:2.6.1.1]](http://www.kegg.jp/dbget-bin/www_bget?ec:2.6.1.1) | 6528.116 | 13135.28 | 15192.07 | 2.01 | 0.02 | 2.33 | | 0.01 |
| Tc09_g030140 | [Anthranilate synthase component I [EC:4.1.3.27]](http://www.kegg.jp/dbget-bin/www_bget?ko:K01657) | 5655.61 | 2604.098 | 2651.337 | 0.46 | 0.01 | 0.47 | | 0.01 |
| Tc02_g016730 | [Tyrosine aminotransferase [EC:2.6.1.5]](http://www.kegg.jp/dbget-bin/www_bget?ec:2.6.1.5) | 12.27432 | 4.937691 | 1.531437 | 0.40 | 0.29 | 0.12 | | 0.03 |
| Tc09_g012270 | [Arogenate dehydrogenase (NADP+) [EC:1.3.1.78]](http://www.kegg.jp/dbget-bin/www_bget?ec:1.3.1.78) | 9.762901 | 8.030735 | 57.45359 | 0.82 | 0.63 | 5.88 | | 0.00 |
| Tc09_g012290 | [Arogenate dehydrogenase (NADP+) [EC:1.3.1.78]](http://www.kegg.jp/dbget-bin/www_bget?ec:1.3.1.78) | 968.643 | 1428.898 | 2348.038 | 1.48 | 0.22 | 2.42 | | 0.00 |
| **^#^**KEGG pathway: **Terpenoid backbone biosynthesis**; [Induced genes (8)](http://www.kegg.jp/kegg-bin/show_pathway?@ko00900/reference%3dwhite/default%3d%23bfffbf/K00626/K01641/K00021/K01597/K01823/K00787/K11778/K15889) and [Repressed genes (3)](http://www.kegg.jp/kegg-bin/show_pathway?@ko00900/reference%3dwhite/default%3d%23bfffbf/K13789/K10960/K15889) | | | | | | | | | |
| Tc01_g019130 | [Acetyl-CoA C-acetyltransferase [EC:2.3.1.9]](http://www.kegg.jp/dbget-bin/www_bget?ec:2.3.1.9) | 50221.05 | 152148.7 | 149377.5 | 3.03 | 0.00 | 2.97 | | 0.00 |
| Tc03_g026590 | [Hydroxymethylglutaryl-CoA synthase [EC:2.3.3.10]](http://www.kegg.jp/dbget-bin/www_bget?ec:2.3.3.10) | 19481.14 | 86973.11 | 100412.3 | 4.46 | 0.00 | 5.15 | | 0.00 |
| Tc02_g012640 | [Hydroxymethylglutaryl-CoA reductase (NADPH) [EC:1.1.1.34]](http://www.kegg.jp/dbget-bin/www_bget?ec:1.1.1.34) | 28164.91 | 137051.6 | 199590.1 | 4.87 | 0.00 | 7.09 | | 0.00 |
| Tc05_g029510 | [Mevalonate kinase [EC:2.7.1.36]](http://www.kegg.jp/dbget-bin/www_bget?ec:2.7.1.36) | 5782.389 | 10408.5 | 9312.315 | 1.80 | 0.00 | 1.61 | | 0.01 |
| Tc03_g030110 | [Phosphomevalonate kinase [EC:2.7.4.2]](http://www.kegg.jp/dbget-bin/www_bget?ec:2.7.4.2) | 5587.363 | 11044.02 | 9519.358 | 1.98 | 0.00 | 1.70 | | 0.01 |
| Tc10_g010950 | [Diphosphomevalonate decarboxylase [EC:4.1.1.33]](http://www.kegg.jp/dbget-bin/www_bget?ec:4.1.1.33) | 16919.77 | 57875.75 | 53947.53 | 3.42 | 0.00 | 3.19 | | 0.00 |
| Tc05_g019150 | [Isopentenyl-diphosphate Delta-isomerase [EC:5.3.3.2]](http://www.kegg.jp/dbget-bin/www_bget?ec:5.3.3.2) | 28084.23 | 59670.05 | 55516.72 | 2.12 | 0.00 | 1.98 | | 0.00 |
| Tc06_g021060 | Top of Form  [Farnesyl diphosphate synthase [EC:2.5.1.1/10]](http://www.kegg.jp/dbget-bin/www_bget?ko:K00787)Bottom of Form | 17864.23 | 59809 | 57336.76 | 3.35 | 0.00 | 3.21 | | 0.00 |
| Tc01_g000540 | [Ditrans,polycis-polyprenyl diphosphate synthase [EC:2.5.1.87]](http://www.kegg.jp/dbget-bin/www_bget?ec:2.5.1.87) | 3539.981 | 7645.911 | 8279.505 | 2.16 | 0.01 | 2.34 | | 0.00 |
| Tc02_g034840 | [Prenylcysteine alpha-carboxyl methylesterase [PCME]](http://www.kegg.jp/dbget-bin/www_bget?ko:K15889) | 859.6745 | 1766.046 | 1886.625 | 2.05 | 0.01 | 2.19 | | 0.00 |
| **^*^**Cacao gene Ids are based on *T*. *cacao* genome (Argout *et al.*, 2010) http://cocoagendb.cirad.fr/gbrowse/cgi-bin/gbrowse/theobroma/  #Click once to follow the pathway. Enzymes marked in green are the differentially expressed based on the homology with transcribed amino acid sequence of the cacao gene.  ^¥^ Click once to follow the gene/enzyme description.  ^±^Fold change highlighted as green indicates induction and blue indicates repression in response to Pmeg/Ppal infection. P-value highlighted as yellow are ≥ 0.05. | | | | | | | | | |
